# Broad scale proteomic analysis of heat-destabilised symbiosis in the hard coral *Acropora millepora*

**DOI:** 10.1038/s41598-021-98548-x

**Published:** 2021-09-24

**Authors:** K. Petrou, B. L. Nunn, M. P. Padula, D. J. Miller, D. A. Nielsen

**Affiliations:** 1 School of Life Sciences, University of Technology Sydney, Sydney, NSW Australia; 2 Department of Genome Sciences, University of Washington, Seattle, WA USA; 3 ARC Centre of Excellence for Coral Reef Studies, James Cook University, Townsville, QLD Australia; 4 Centre for Tropical Bioinformatics and Molecular Biology, James Cook University, Townsville, QLD Australia

**Keywords:** Molecular biology, Physiology, Climate sciences, Ecology, Environmental sciences

## Abstract

Coral reefs across the globe are threatened by warming oceans. The last few years have seen the worst mass coral bleaching events recorded, with more than one quarter of all reefs irreversibly impacted. Considering the widespread devastation, we need to increase our efforts to understanding the physiological and metabolic shifts underlying the breakdown of this important symbiotic ecosystem. Here, we investigated the proteome (PRIDE accession # PXD011668) of both host and symbionts of the reef-building coral *Acropora millepora* exposed to ambient (~ 28 °C) and elevated temperature (~ 32 °C for 2 days, following a five-day incremental increase) and explored associated biomolecular changes in the symbiont, with the aim of gaining new insights into the mechanisms underpinning the collapse of the coral symbiosis. We identified 1,230 unique proteins (774 host and 456 symbiont) in the control and thermally stressed corals, of which 107 significantly increased and 125 decreased in abundance under elevated temperature relative to the control. Proteins involved in oxidative stress and proteolysis constituted 29% of the host proteins that increased in abundance, with evidence of impairment to endoplasmic reticulum and cytoskeletal regulation proteins. In the symbiont, we detected a decrease in proteins responsible for photosynthesis and energy production (33% of proteins decreased in abundance), yet minimal signs of oxidative stress or proteolysis. Lipid stores increased > twofold despite reduction in photosynthesis, suggesting reduced translocation of carbon to the host. There were significant changes in proteins related to symbiotic state, including proteins linked to nitrogen metabolism in the host and the V-ATPase (-0.6 fold change) known to control symbiosome acidity. These results highlight key differences in host and symbiont proteomic adjustments under elevated temperature and identify two key proteins directly involved in bilateral nutrient exchange as potential indicators of symbiosis breakdown.

## Introduction

Many reef building corals live in symbiosis with photosynthetic microalgae from the family *Symbiodiniaceae*, a tightly regulated relationship that has been pivotal to their success in nutrient-poor tropical waters^1^. This mutualistic partnership is fine-tuned to exist under relatively stable temperature conditions, which means that tropical reef building corals live close to their thermal threshold, where even moderate increases in water temperature can reduce coral fitness^2,3^. If thermal stress is prolonged, destabilisation and collapse of this symbiosis can ensue via a phenomenon known as coral bleaching (a whitening of the coral tissue due to the loss of symbionts and pigments). Because of this inherent sensitivity to small rises in seawater temperature, climate change-induced marine heat waves and warmer than average waters are having catastrophic impacts on coral reef ecosystems^4^. In the wake of recent recurrent mass bleaching events^5^, confidence in the predictions of ecosystem-wide warming episodes occurring with greater frequency and intensity has strengthened^6^. While the environmental cues and dominant physiological processes involved in the bleaching response are well-defined, gaps in knowledge about the molecular mechanisms that underpin the stress response to elevated temperature in corals exist. To that end, recent decades have seen a multitude of studies investigating physiological processes that sustain the homeostasis of the coral symbiosis, and others aimed at detecting the drivers that underpin the breakdown of this relationship.

For the dinoflagellate symbiont under bleaching conditions, studies focussed on photophysiological responses have documented consist downturn in photosynthetic efficiency^7–13^ and often some level of photoinhibition or damage to the symbiont’s photosynthetic machinery^14–17^. However, these observations are complicated by varying levels of temperature resistance across the various *Symbiodiniaceae* species^16,18^. For the less studied coral host, fewer consistent patterns have emerged. Reports on key physiological responses by the host to elevated temperature include changes to respiration rate and metabolism^19–22^, loss in cell adhesion properties^23,24^ and increased antioxidant production^11–13^. Until recently, however, there has been minimal investigation into the combined and concurrent metabolic changes in both partners to uncover how this relationship goes awry when things start to heat up.

Recent analytical advancements and the broader uptake of ‘omics approaches have accelerated our understanding of the metabolic maintenance and regulation of this important partnership. Transcriptomic^25–27^ and proteomic^28–30^ studies comparing symbiotic and aposymbiotic *Aiptasia* have uncovered symbiosis-specific genes, proteins and metabolic pathways, highlighting nutrient exchange and transport processes^26,28,30^, as well as protein phosphorylation status^31^, as notable indicators of symbiotic state. Investigations into the cellular response of the cnidarian host (*Aiptasia* or coral) and/or its dinoflagellate symbiont to thermal stress have also been targeted with transcriptomic^22,32^, proteomic^29,33,34^ and metabolomic^35–37^ analyses. Common responses to heat stress, such as protein folding and increased antioxidant expression, have been reported in the transcriptome and proteome of an *Aiptasia*-algal symbioses under acute thermal stress^38^, the proteome of *Pocillopora acuta*^34^ and the heat stressed transcriptome of *Acropora aspera*^39^. Transcriptomic studies on corals have revealed strong differential expression in the energy pathways of both the host and symbiont^22^ and stress response genes to be more responsive^32^ and immune suppression longer lasting^40^ in the host than in the symbiont. Heat-induced changes in the *Aiptasia*-symbiont metabolome include increased catabolism of lipid stores in both partners and accumulation of free fatty acids, glucose and lipogenesis intermediates in the symbiont^35,36^. The multiplicity of these findings, while likely the result of differences in experimental conditions, symbioses, species, and methodologies, highlight the complexity in uncovering concurrent metabolic changes occurring in two phylogenetically distant organisms living as one holobiont.

Not with standing the significant advances these ‘omic studies have generated towards our understanding of the metabolic processes underpinning cnidarian-algal symbioses, very few studies^34^ have used proteomics to examine both the coral host and symbiont responses to thermal stress by separating a single holobiont system into its host and symbiont constituents, allowing direct comparison of responses in the two partners. Yet, such approach is imperative if we are to elucidate the interconnected response patterns that underlie the maintenance and degradation of this important relationship. Mass spectrometry-based shotgun proteomics provides a highly sensitive and direct approach for detecting changes in the cellular machinery underlying adjustment to temperature stress.

In this study, we explore the proteomes of the host and symbionts concurrently in the important reef-building coral *Acropora millepora* exposed to elevated temperature. Using MS-based proteomics, we uncover physiological processes in the coral host and symbionts that are affected during the onset of bleaching and explore associated biomolecular changes using single-cell FTIR. Here, we reveal molecular-level insights into the mechanisms that drive the collapse of the coral symbiosis and subsequent bleaching.

## Results and discussion

### Coral physiology in response to elevated temperature

**Figure 1:**
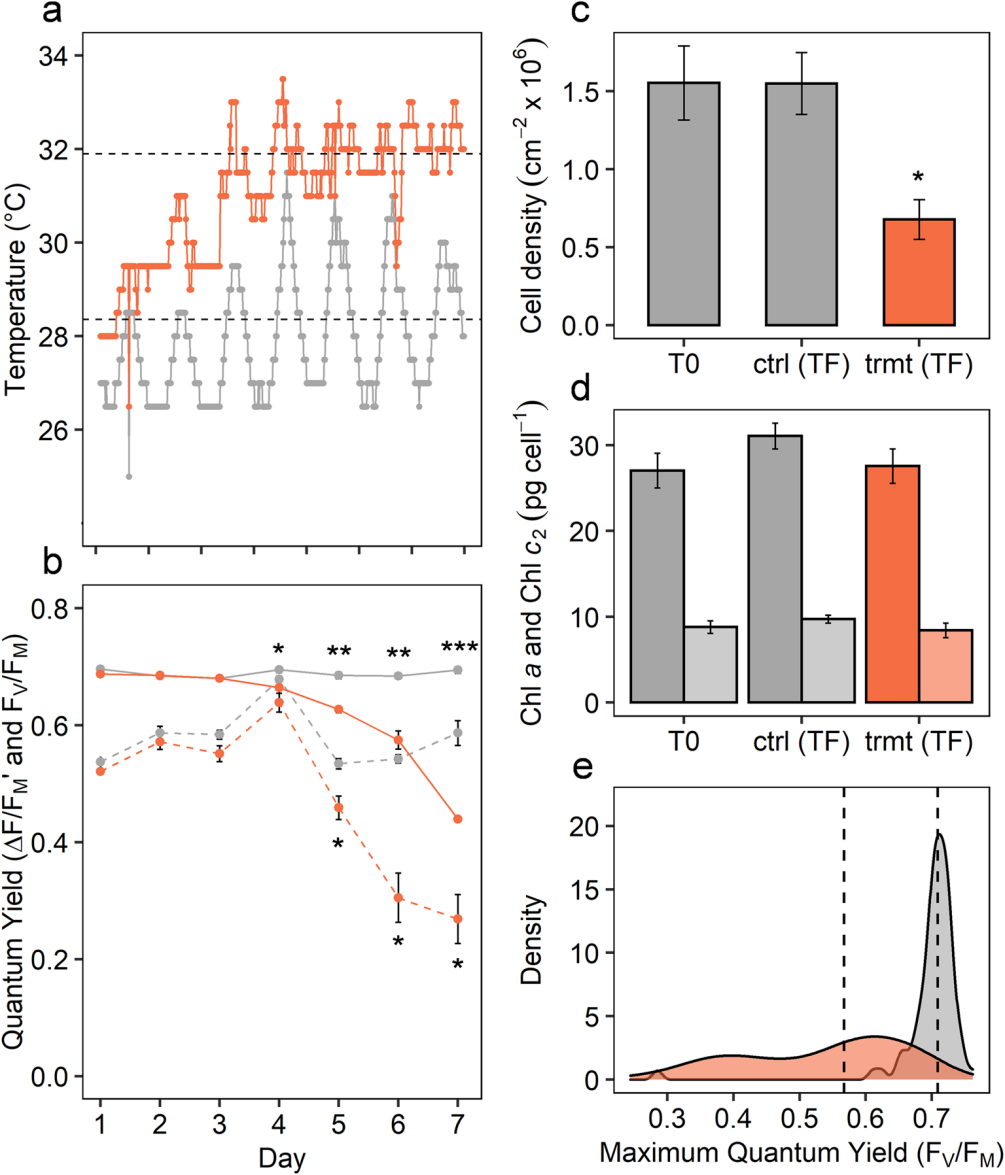
Temperature and general physiology of *Acropora millepora* and its symbionts under control (grey) and treatment (orange) conditions: (**a**) logged temperature in coral tanks over experimental period, (**b**) maximum (F_V_/F_M_) and effective (ΔF/F_M_′) quantum yield of PSII of coral colonies, (**c**) cell density at initial (T0), and final (TF) time points, (**d**) chlorophyll *a* and *c*_*2*_ per cell at T0 and TF, (**e**) density plot of single-cell F_V_/F_M_ measurements of symbionts extracted from *Acropora millepora* colonies at TF. Data (**b, c**, **d**) represent mean values ± standard error, and dotted lines (**a** and **e**) indicate mean. Asterisks denote significant differences between control and treatment at * < 0.05, ** < 0.01, *** < 0.001.

Sustained declines in photosynthetic health and symbiont density are well-defined characteristics of coral bleaching^41^. Consistent with previous studies^10,11^, the photosynthetic health of the coral symbionts, measured as dark-adapted quantum yield of PSII (F_V_/F_M_), decreased towards the end of the temperature ramping period (from day 4), declining further over the following three days (rmANOVA; F_6,39_ = 129.9, *P* < 0.001; Fig. 1a). Similarly, midday measurements of effective quantum yield of PSII (ΔF/F_M_)′ showed continuous decline under elevated temperature from day 5 (rmANOVA; F_6,39_ = 21.85, *P* < 0.001; Fig. 1b). After two days at target temperature (mean 31.9 °C), mean symbiont cell density had declined significantly (t = 3.95, df = 3, *P* = 0.028) to less than 50% of control corals (Fig. 1c), indicating bleaching via symbiont loss, but with no change in chlorophyll *a* or *c2* per cell (Fig. 1d). Congruent with these data, single-celled measurements of F_V_/F_M_ showed a significant shift in population distribution (KS; D = 0.797, *P* < 0.001), from a tight cluster of healthy endosymbiotic (*in-hospite*) cells (median = 0.709) to a broad spread in F_V_/F_M_ (median = 0.567) of the *in-hospite* cells under elevated temperature (Fig. 1e).

### Biomolecular profile of the symbiont using FTIR microspectroscopy

**Figure 2:**
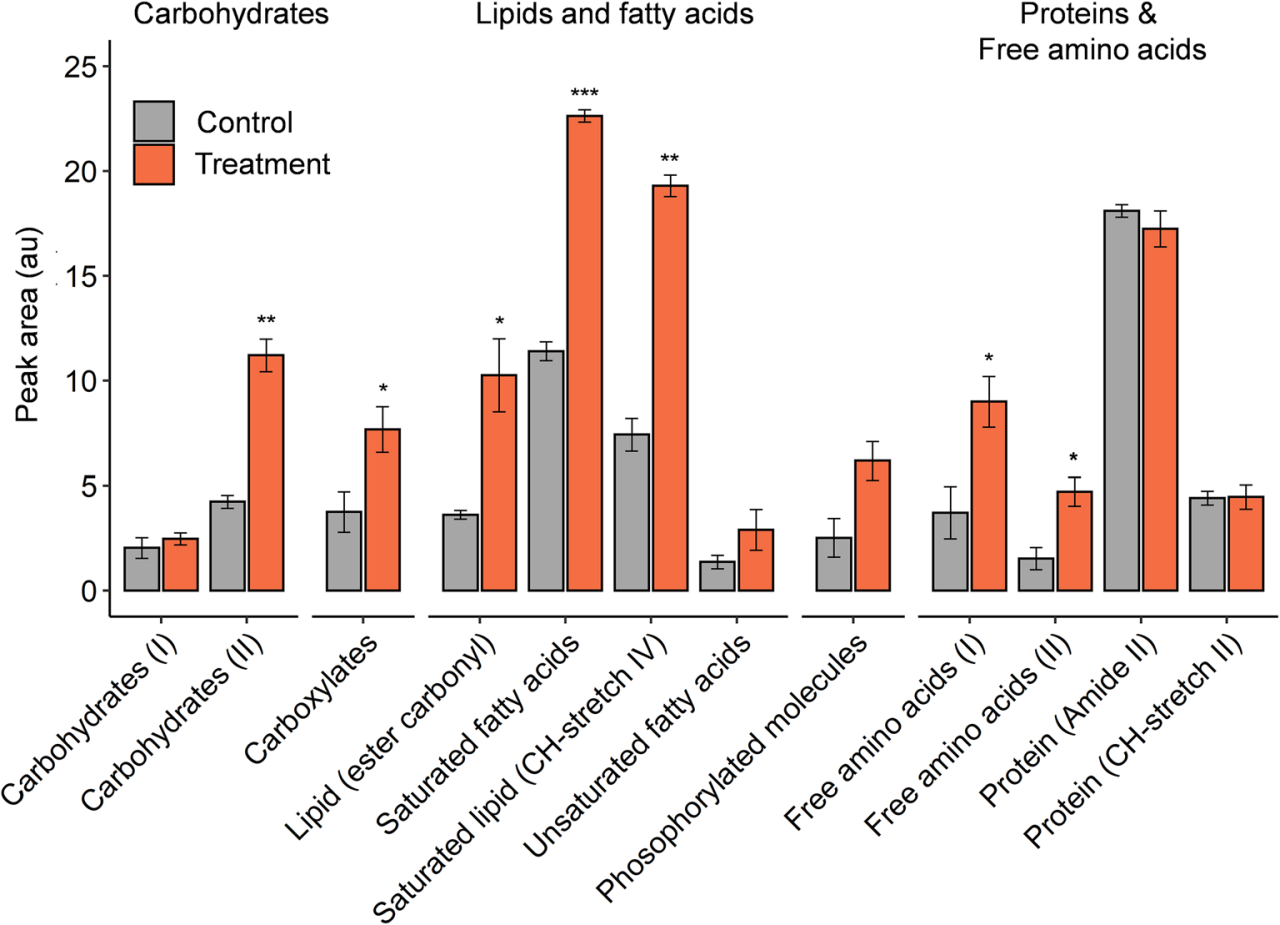
Macromolecular content of control (grey) and heat-treated (orange) symbionts measured by FTIR microspectroscopy. Data represent mean relative content of detected biomolecules ± standard error (*n* = 4). Asterisks denote significant differences (paired t-test) between control and treatment at * < 0.05, ** < 0.01, *** < 0.001.

There were significant differences in biomolecular content of coral symbionts with elevated temperature (Fig. 2) with increases in the relative concentrations of saturated fatty acids (t = − 15.65, df = 3, *P* < 0.001), saturated lipids (t = − 10.53, df = 3, *P* = 0.002), ester carbonyl (t = − 4.19, df = 3, *P* = 0.025), carboxylates (t = − 3.59, df = 3, *P* = 0.037), and free amino acids I (t = − 4.31, df = 3, *P* = 0.023) and II (t = − 4.7013, df = 3, *P* = 0.018), consistent with the findings of Petrou et al.^10^. In contrast to this previous study however, there were no declines in protein-related biomolecules (Amide II, CH-stretch II) and an increase in Carbohydrate II (t = − 11.50, df = 3, *P* = 0.001). The overall accumulation of energy stores (lipid and carbohydrate), despite the downregulation of energy production (photosynthesis), could be indicative of reduced translocation of metabolites to the host.

### Overview of proteomic analysis

**Figure 3:**
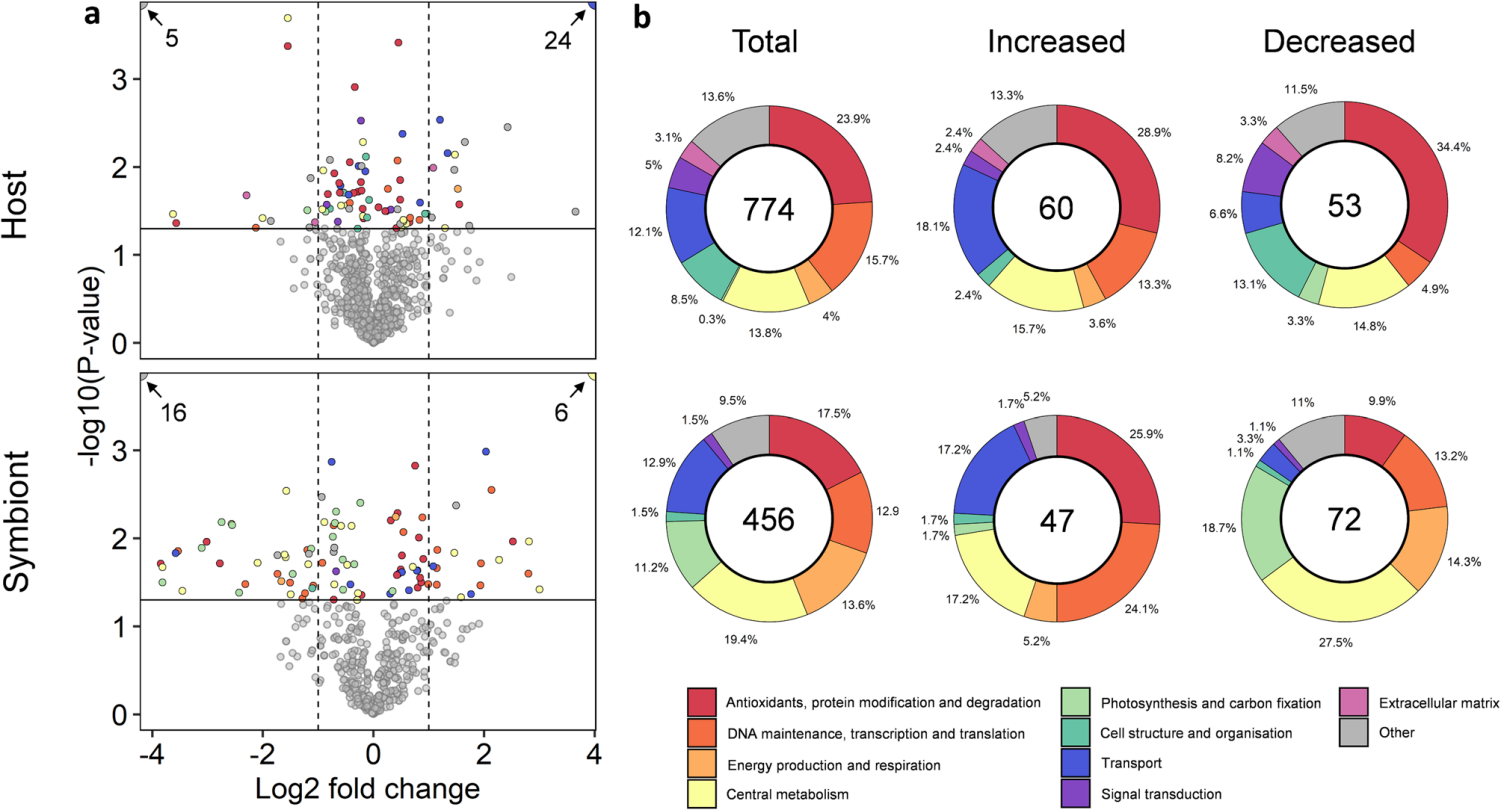
Overview of proteomic analyses: (**a**) Funnel plot of log2 fold change vs −log10 *p*-value of detected proteins and (**b**) total number and proportional changes in differentially expressed proteins grouped at the level of biological and molecular function (GO-term classification) for the host and symbiont proteomes in response to elevated temperature. Vertical dashed lines indicate a log2 fold-change of 1, solid horizontal line indicates significance value (−log10 *p*-value < 0.05). Numbers inside circles indicate abundance of total and differentially expressed proteins detected in host and symbiont. Colours indicate functional grouping of proteins.

A total of 1,230 unique proteins were identified (774 host and 456 symbiont) at a false discovery rate ≤ 0.01. Of those, 107 were significantly increased in abundance and 125 decreased in abundance (see Supplementary Tables 2 [host] and 3 [symbiont] for all differentially abundant proteins), while the remainder (998) displayed no significant change in abundance between control and treatment (see supplementary data file). Of the 113 proteins that were identified to be differentially expressed between the control and treatment in the host (Fig. 3a), 37 proteins had log2 fold changes ≥ 1 (increased abundance), while 17 proteins yielded log2 fold changes ≤ − 1 (decreased abundance). In the symbiont, of the 119 proteins that were significantly increased or decreased in abundance, 24 were identified to have a fold change of ≥ 1 and 48 proteins had a fold change of ≤ − 1 (Fig. 3a). Given that the total number of proteins within a given metabolic pathway were often low, we assessed the overall reorganisation of the host and symbiont proteomes in response to thermal stress at the level of biological and molecular function, based on groupings of GO-term classifications (Fig. 3b; Supplementary Tables 2 and 3).

### Functional analysis of the host proteome response to elevated temperature

#### Host: antioxidant function, protein modification and degradation

**Figure 4:**
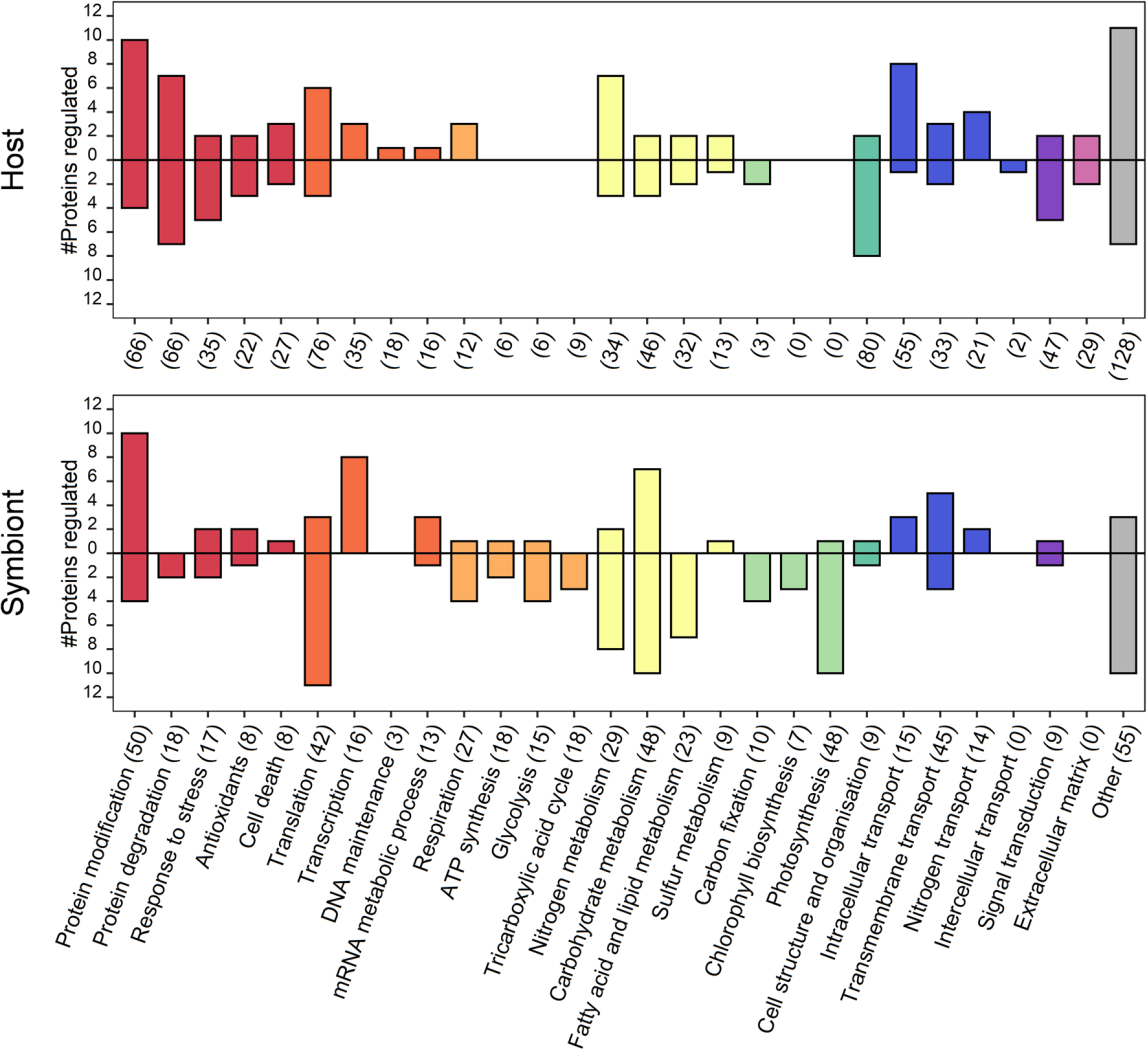
Number of differentially expressed proteins detected in the host and symbiont of *Acropora millepora* grouped by sub-categories. Positive bars show the number of proteins that increased in abundance, while negative bars show the proteins that decreased in abundance with elevated temperature. Numbers in parentheses on the x-axis indicate the total (including unchanged) number of proteins detected for each sub-category. Colours represent major functional groups, as described in the legend of Fig. 3.

The greatest number of differentially abundant proteins in the host were associated with protein modification and degradation (Fig. 4). Of the 66 linked to modification processes, 14 were differentially expressed. Most of the proteins that showed an increase in abundance in this groups were heat shock proteins (HSPs), other chaperones and proteins involved in folding, quality control, and proteolysis. Specifically, we detected SGT1 homolog A, possibly involved in the ubiquitination of HSP90 client proteins and an Activator of 90 kDa HSP ATPase homolog 1, a co-chaperone of HSP90AA1. As expected, there were significant increases in HSP90 kDa (0.65 FC) and HSP90-alpha (0.48 FC), consistent with temperature shocked anemones and corals^29^. Also, we detected increases in two endoplasmic reticulum (ER) disulfide-isomerases (A4, 0.45 FC; A3, 0.21 FC) and the chaperone protein BiP (0.94 FC), which are important for the identification and translocation of misfolded proteins^42^. BiP is an HSP70 molecular chaperone located in the lumen of the ER and, while abundant under all growth conditions, it is strongly induced under conditions that lead to the accumulation of unfolded polypeptides in the ER^43^.

Of the 66 proteins detected that were associated with proteolytic function (protein degradation), seven were increased in abundance, including the metalloprotease component of the 26S proteasome (1.55 FC), a peptidase involved in the regulation of intracellular protein levels and selective removal of damaged or incorrectly folded proteins^44,45^. Previous studies have found this proteasome to be responsive to oxidative stress, becoming elevated in both anemones^29^ and foraminifera^46^ under thermal stress. Counter to this, we detected a decrease in the Proteasome subunit alpha type-6 (-0.71 FC), a component of the 20S core proteasome complex involved in the proteolytic degradation of most intracellular proteins. This complex plays numerous essential roles within the cell by associating with different regulatory particles including participating in ATP-dependent degradation of ubiquitinated proteins. These differential changes to proteolytic regulation indicate a change in the hosts ability to maintain proteostasis via efficient protein quality control.

We detected seven ‘response to stress’ proteins that were regulated in the host. Among them, Heme-binding protein 2 (− 1.55 FC), a protein that can promote mitochondrial permeability and facilitate necrotic cell death under different types of stress conditions, decreased in abundance^47^. We also detected a Transmembrane emp24 domain-containing protein 4, a protein involved in ER stress response that may play a role in the regulation of heat-shock response and apoptosis^48^. Taken together, the increase in chaperones, changes to proteolytic function and decrease in proteins able to control necrotic cell death suggests a loss in regulatory processes essential to general proteostasis.

Reactive oxygen species (ROS) are a natural by-product of aerobic metabolism, but their over-production has been implicated in the bleaching response to thermal stress^2,49,50^. We detected 22 proteins related to antioxidant function in the host, of which only five were identified to have significantly changed abundances relative to the control (Fig. 4). Well studied antioxidants, like Superoxide dismutase, Catalase and Thioredoxins, were detected, but were not differentially abundant (see Supplementary data file). Instead, temperature stress resulted in the unique detection of Glutathione S-transferase (undetected in control samples), which catalyses the binding of the reduced form of glutathione (GSH) to xenobiotic substrates for the purpose of detoxification. The host also increased synthesis of Methionine adenosyltransferase 1 (MAT 1), a protein in the methionine cycle responsible for the synthesis of S-adenosyl methionine (SAMe). SAMe is a primary methyl donor in eukaryotic cells and an important precursor for GSH. This increase in MAT in the cnidarian host is consistent with changes observed in heat shocked *Aiptasia*^29^, and in *Acropora* spp. under a variety of stressors^51,52^ and given the intermediary link between MAT and GSH, it is possible that an increase in MAT, and thus SAMe levels, may be complicit in supporting the antioxidant system.

For a range of marine invertebrates, the thioredoxin-peroxiredoxin system has been implicated in scavenging hydrogen peroxides, but its activity is not often measured^53^. Here we detected an increase in peroxiredoxin-4 (0.48 FC), a thiol-specific peroxidase that catalyses the reduction of hydrogen peroxide (H_2_O_2_) and organic hydroperoxides to water and alcohols, respectively. Surprisingly however, both peroxiredoxin-5 (− 0.23 FC) and peroxiredoxin-6 (− 0.28 FC), which also function in cell protection against oxidative stress by detoxifying peroxides, decreased in abundance following heat stress. Together, these data indicate that although the response of the coral host to thermal stress involves upregulation of several components of the antioxidant system, it may also simultaneously have reduced levels of other proteins required for detoxification.

Elevated levels of three proteins annotated in the cell death category (of a total of 27), including a caspase-7-related protein, again suggest that the host may have suffered extensive oxidative damage. Caspase-7 (0.23 FC) is an executioner caspase, relatives of which have been implicated in apoptosis in corals^54,55^. Executioner caspases are constitutively synthesised but normally activated (by cleavage) only after cells have sustained irreversible damage. The heterogeneity in the responses of tissues or cell-types to temperature-induced oxidative stress could potentially explain the apparent contradictions observed in these data. Symbionts reside exclusively in the gastrodermal (= endodermal) cells of the coral, and it is these, rather than the ectodermal cells, which are likely to be the immediate victims of oxidative stress caused by the symbionts.

#### Host: central metabolism and calcification

Cell homeostasis relies on balancing energy requirements (e.g. ATP and NADPH) with the production and utilisation of metabolites, which means that shifts in metabolic function are needed to acclimate to environmental change. Of the 149 host proteins identified that were classified into the functional group ‘central metabolism’, 23 were present at different levels in heat-treated and control samples (Fig. 4).

*Nitrogen metabolism in the host* Amongst the differentially abundant proteins associated with nitrogen metabolism under heat stress were Glutamate dehydrogenase (GDH; 0.54 FC), which increased in abundance, and Glutamine synthetase (GS; − 1.55 FC), which decreased in abundance. Increased GS activity has been reported as a characteristic of symbiotic cnidarians^28,56,57^ and hypothesised to serve as a mechanism for imposing nitrogen limitation on the endosymbiont, restricting its growth^30^. As ammonium assimilation via the high-affinity GS system is energetically costly, decreased levels of this protein reflect metabolic expediency during the collapse of the symbiotic state. To compensate for the decline in GS activity, increased levels of the low affinity, but energetically inexpensive, GDH protein are presumably required to maintain cellular homeostasis during symbiosis breakdown. Note that the dramatic decline in the level of Phosophoserine aminotransferase (FT − 3.63; the most reduced of all host proteins) is also consistent with the host transitioning away from reliance on ammonium assimilation via the GS pathway^58^. These results are supported by a recent study on the heat stress response in the coral *Stylophora pistillata*^22^, in which gene expression of GS decreased and the catabolic version of GDH increased, driving amino acid degradation likely to meet the coral’s increased energy requirements (increased respiration). In line with these findings, we measured increased proteolysis and detected increases in proteins associated with respiration (see Supplementary Results and Discussion). If, as proposed by Rädecker et al*.* (2021), translocation of carbon from the symbiont is insufficient to meet host demand under elevated temperatures, then the host would be forced to degrade its own energy stores. Our study detected an increase in proteins involved in the degradation of lipids, proteins and fatty acids, all of which could be attributed to a direct response to energy limitation in the host.

*Carbohydrate and lipid metabolism in the host* Although few changes in abundance were observed in proteins associated with carbohydrate or lipid metabolism (5/46 and 4/32, respectively; see Fig. 4), most of these reflect a shift from reliance on translocated photosynthate to storage lipid breakdown. Cnidarians are amongst the few animal groups in which the glyoxalate pathway is present; by enabling the product of beta-oxidation of lipids (acetyl CoA) to support gluconeogenesis, the combined actions of isocitrate lyase and malate synthase in the glyoxylate cycle facilitate breakdown of storage lipids, enabling their use for energy production. In heat stressed *Porites asteroides*^59^, gene expression of isocitrate lyase increased in abundance but expression of malate synthase did not, and the same scenario was seen at the proteomic level in our *Acropora millepora* data. The observed decline in the level of Fumarylacetoacetate hydrolase (FAH) domain-containing protein 2 can also be rationalised in terms of operation of the glyoxalate pathway, as many members of the FAH domain-containing protein family have oxaloacetate hydrolase activity^60^, which would interfere with operation of the glyoxalate pathway. Also consistent with a metabolic switch from reliance on carbohydrate metabolism was an observed reduction in the levels of UTP–glucose-1-phosphate uridylyltransferase (− 0.91 FC) after heat stress. This enzyme is involved in glycogen biosynthesis and is upregulated during the establishment of symbiosis in cnidarians^61^. The increased abundance of a Phospholipase B-like 1 (0.54 FC; Fig. 4), which is capable of releasing fatty acids from phospholipids, and the electron transfer Flavoprotein subunit alpha (1.29 FC), a protein required for beta-oxidation of fatty acids and normal amino acid metabolism^62^, together with decreased abundance (twofold) of an Acetyl-CoA synthetase 2-like protein, the roles of which include fatty acid biosynthesis from carbohydrates, provides further support for the hypothesis that the host was mobilising lipid stores to compensate for decreased availability of algal photosynthate.

*Calcification and symbiosis* Two of the three carbonic anhydrases identified significantly decreased in abundance in the heat-treated corals (-1.2 FC), a response consistent with previous work^63^. In reef building corals, carbonic anhydrases (CAs) play two key roles in calcification of the skeleton: supplying dissolved inorganic carbon (DIC) for calcium carbonate precipitation, and the removal of carbonic acid from the precipitation site^64^. Thus, a reduction in this enzyme suggests significant disruption to calcification processes under thermal stress^63,65^. Also consistent with suppression of calcification by heat stress, lower levels (− 2.30) of an acidic protein that is a component of the skeletal organic matrix^25,66^ were detected after heat stress.

Of the total 111 proteins classified as being involved in intracellular, transmembrane and nitrogen transport, 19 differed in abundance following heat treatment (Fig. 4). Of these, the decreased abundance of a V-ATPase subunit A (-0.60 FC) protein may be directly relevant to the collapse of the symbiosis. V-ATPases are responsible for acidifying intracellular compartments and in corals, by acidifying the symbiosome, a V-ATPase has been shown to both promote symbiont photosynthesis, essentially acting as a component of a carbon-concentrating mechanism (CCM), and to facilitate translocation of photosynthetic products^67^. Therefore, decreased levels of this protein will result in reduced photosynthetic exchange between partners, as both algal photosynthesis and translocation of photosynthate will decline.

#### Host: cell structure and organisation

More than 80 proteins associated with cell structure and organisation were detected in the coral host proteome, 10 of which were differentially expressed (Fig. 4). Actin, a principal constituent of the microfilament network and a key component of muscle fibres, is one of the most abundant proteins in eukaryotic cytoskeletons^68^. Several proteins that have known roles in the binding and assembly of actin filaments decreased in abundance in this study, including coactosin-like protein (− 0.79 FC), filamin-A (− 0.14 FC), radixin (− 0.08 FC) and alpha-adducin (− 0.15 FC). There were also decreases in proteins contributing to actin organisation, cell shape and microtubule microstructural dynamics in the cytoskeleton, namely amplaxin (− 0.45 FC) and EBF3 (− 0.54 FC). Actin filaments are known to be sensitive to oxidative damage^68^, and similar negative effects of elevated temperature have been observed in both anemones^29^ and foraminifera^46^. Notably, concomitant with a decline in actin-related proteins, we detected an increase in the cell membrane-cytoskeletal-associated F-actin-uncapping protein LRRC16A (0.93 FC), which plays a role in the disassembly of actin filaments (Fig. 4), corroborating increased actin breakdown. The decline in the structural and cytoskeletal proteins and increase in protein disassembly in this study are consistent with previous work that has shown loss in host tissue integrity with thermal stress^23,24^ and provides further evidence of oxidative damage in the host, suggesting significant cell reorganisation and possibly impaired control over cell structure mechanisms.

Of the 29 proteins associated with the host extracellular matrix, Matrilin-2, a protein which may play a role in adhesion or anchoring of filaments increased in abundance (1.08 FC). Proteins strongly decreased in abundance included integrin alpha-6 (VLA-6), which plays a role in cell adhesion processes (− 1.06 FC). The changes in adhesion proteins and matrix assembly processes suggest that coral tissue integrity may be compromised by heat stress, and together with the overall degradation of adhesion proteins, may be the host’s way of encouraging symbiont expulsion.

### Functional analysis of the symbiont proteome to elevated temperature

#### Symbiont: antioxidant function, protein modification and degradation

Thermal stress in algae can lead to over-production of ROS via photosystem malfunction, resulting in denaturation of cellular proteins and their subsequent degradation^49^. Of the 50 proteins detected likely to be involved in protein modification, fourteen were differentially expressed under elevated temperature (Fig. 4). As found in the host, the largest group of proteins to increase in abundance were heat shock proteins (HSPs). Heat shock like 85 kDa protein, which is linked to ATPase activity, increased 0.9-fold, while the fold increases in other HSPs (HSP90, HSP70-14, HSP70, HSP70 (DnaK) and HSP60-2) ranged between 0.43 and 0.82. A small number (three of eight;) of proteins involved in detoxification and stress responses in the symbiont changed in abundance with heat treatment (Fig. 4). Peroxiredoxin-2 and -5 increased in abundance 0.80 and 0.84 FC, respectively, whereas phosphoglucan water dikinase, a protein important for starch degradation in the chloroplast, was significantly depleted (− 3.85 FC) after heat treatment. Several proteins associated with death and senescence (Dipeptidyl peptidase 8, cysteine proteinase SAG39 [− 0.72 FC], Serine/threonine-protein phosphatase 5 [− 3.02 FC] and DAP kinase 3 [− 0.22 FC]) also decreased in abundance.

While the strong expression of chaperones is consistent with previous studies on coral symbionts under thermal stress^29,32,33,69^, the limited antioxidant response and lack of any evidence for cell death or protein degradation were unexpected, and consistent with the idea that symbionts mount a damage control response but remain viable under the type of heat-stress imposed here. Furthermore, we found no change in relative protein content in the biomolecular data (Fig. 2), consistent with the absence of increased protease levels. In line with previous studies^11–13,70^, the relatively slow, yet prolonged, thermal stress treatment reduced photosynthetic performance but may not have induced heavy oxidative stress and subsequent deterioration of symbiont cells.

#### Symbiont: central metabolism

*Nitrogen metabolism* Tight regulation of nitrogen cycling between the host and symbiont is often considered central to the evolutionary success of the coral symbiosis^56,71,72^. Of the 29 symbiont proteins detected that were nominally associated with nitrogen metabolism, ten were affected by elevated temperature and the majority of these decreased in abundance (Fig. 4). Both methionine synthase (MS), which methylates homocysteine to regenerate methionine, and type-3 glutamine synthetase (GS), an enzyme essential in ammonium assimilation and glutamine biosynthesis^73^ were undetectable following heat-treatment. While these changes may indicate nitrogen limitation, it must be noted that two other proteins also classified as GS and with higher spectral counts did not change significantly following the treatment (see Supplementary Data File), suggesting glutamine biosynthesis may not have been severely affected in the symbiont; a finding consistent with symbionts of *Acropora aspera*^32^ and *Stylophora pistillata*^22^ under thermal stress.

*Carbohydrate metabolism* A total of 17 symbiont proteins classified as associated with carbohydrate metabolism changed during the heat stress experiment. Proteins that increased in abundance included Glycerol-3-phosphate dehydrogenase (GPDH) (0.61 FC), an important link between carbohydrate and lipid metabolism. GPDH catalyses the reduction of NADH and dihydroxyacetone phosphate (DHAP) to form NAD^+^ and Glycerol-3-phosphate (G3P), an intermediary metabolite that connects multiple metabolic pathways such as glycerolipid synthesis, glycolysis and gluconeogenesis. Overexpression of GPDH in the marine diatom *Phaeodactylum tricornutum* was shown to result in significant increases (6.8-fold) of glycerol and a 60% increase in neutral lipid content^74^. Thus, the increase in GPDH here could equate to increase in G3P and therefore more glycerol and lipid, a response commonly observed in the coral symbionts under temperature stress^10,35,37,75^.

The heat-treated proteome also showed evidence of increased carbohydrate production, consistent with the FTIR biomolecular data (Fig. 2). UDP-sugar pyrophosphorylase (0.71 FC), which converts sugar-1-phosphate into UDP-glucose, and UDP-glucose 6-dehydrogenase (2.81 FC), responsible for the interconversion of UDP-glucose and UDP-glucuronate, an intermediate in polysaccharide biosynthesis including that of hemicellulose and pectin in plants^76^, were both significantly more abundant. UDP-sugars are the main precursors of biomass production and primary metabolites in plants (e.g. sucrose, cellulose, hemicellulose and pectin), as well as glycoproteins and glycolipids^77^, and starch synthesis in dinoflagellates is thought to involve the incorporation of UDP-glucose by granule-bound starch synthase^78^.

We also detected increases in phosphoenolpyruvate carboxykinase (PEPCK; 0.79 FC), which is part of the gluconeogenesis pathway (the production of glucose from non-carbohydrate compounds). In addition to the loss of two pyruvate kinases (PKLR, PK), co-associated with the glycolysis pathway (see Supplementary Results and Discussion), there was a decrease in the abundance of proteins associated with CoA transferase processes in carbohydrate metabolism. CaiB/b (− 3.82 FC) and pentafunctional AROM polypeptide (− 0.59 FC) both decreased and there was decreased abundance of a glycogen branching enzyme (1,4 alpha; − 2.1 FC), a protein involved in glycogen biosynthesis and accumulation^79^. In direct contrast with the animal host, the decreased abundance of Isocitrate lyase 1 (− 0.28 FC) detected in the symbiont presumably limits the breakdown of storage lipids, thereby eliminating a potential nutrient source via storage lipid mobilisation^80,81^. The loss of glycolytic enzymes combined with declines in proteins involved in glycogen biosynthesis, suggest a slowing in the symbiont’s energy production. Both lipid accumulation and higher carbohydrate content that was observed in the FTIR data (Fig. 2) are congruent with the increase in UDP sugar production for sucrose, cellulose or glycolipid synthesis observed in the proteome.

*Fatty acid and lipid metabolism* All six of the 23 detected proteins associated with fatty acid and lipid metabolism that were differentially regulated, decreased in the symbiont proteome (Fig. 4). The symbiont decreased soluble inorganic pyrophosphatase 1 to below detection level; a protein which catalyses the conversion of pyrophosphate into phosphate ions, releasing energy that can be used to catalyse otherwise unfavourable chemical reactions. This mechanism is particularly important for the activation of fatty acids for beta-oxidation^82^. There was also a strong decline in the abundance of acetyl-CoA carboxylases (− 1.58 to − 1.61 FC), which provide the malonyl-CoA substrate for fatty acid biosynthesis^83^. Congruent with the above, there was a decrease in glycerol-3-phosphate acyltransferase (GPAT; -1.5 FC), the rate-limiting enzyme in the de novo pathway of glycerolipid synthesis, which plays a pivotal role in the regulation of triglyceride (TAG) and phospholipid synthesis^84^. Together with the downregulation of ATP-citrate synthase subunit 1 (− 0.89 FC), a protein that catalyzes the formation of cytosolic acetyl-CoA that is mainly used for the biosynthesis of fatty acids and sterols, these changes to the symbiont proteome signal a decline in fatty acid and lipid biosynthesis. Despite this apparent downregulation in lipid production, we observed an accumulation of lipid in the cell (Fig. 2), an inconsistency that may be explained by reduced fatty acid translocation to the host and increased glycerol production within the cell.

#### Symbiont: photosynthesis and carbon fixation

**Figure 5:**
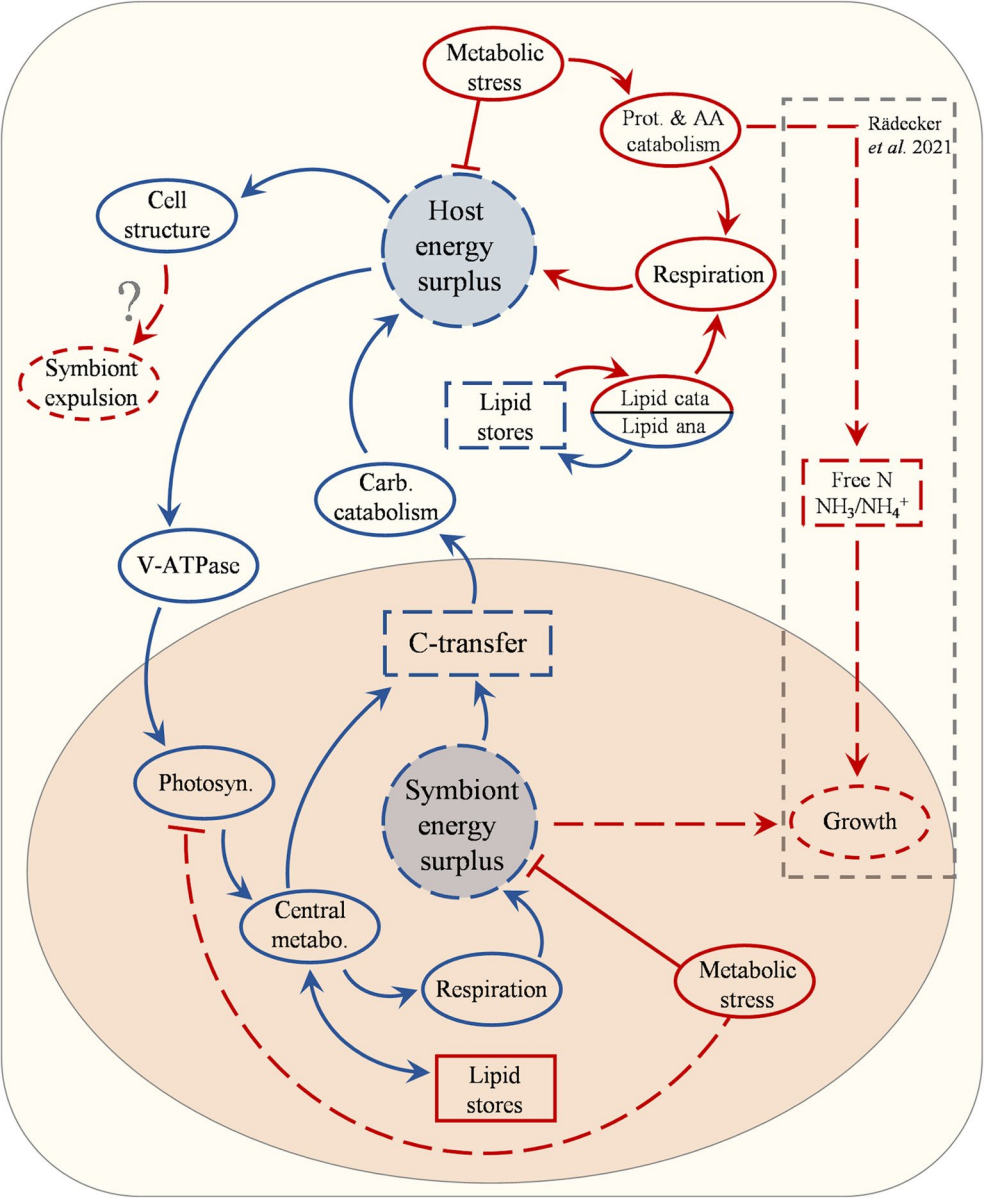
Hypothesised mechanism for coral symbiosis destabilisation based on proteomic analysis of *Acropora millepora* and its associated symbionts under thermal stress. Elevated temperature induces metabolic stress in the host causing protein destabilisation and increase in respiration. Protein degradation leads to the release of free amino acids, which together with lipids, are degraded to support the increased respiratory demand. Due to less energy available, the energetically expensive symbiosome membrane protein V-ATPase declines, reducing inorganic carbon availability to the symbiont, thus reducing photosynthetic activity. This leads to reduced activity in the central metabolic processes in the symbionts (carbohydrate and lipid metabolism). The overall lowered metabolic activity of the symbiont together with increased housekeeping requirement, due to increased temperature, reduces the pool of surplus energy and excess carbon available for transfer to the host, which subsequently experiences further energy limitation. The evidence obtained in this study supports the hypothesis presented in Rädeker et al*.* (2021), whereby the reduction in carbon transfer is the mechanism underlying the breakdown of the symbiotic relationship in corals. While we did not obtain direct evidence for the process of increased growth in the symbionts from availability of free nitrogen as proposed by Rädecker et al*.* (2021), the mechanism fits the scheme suggested here and would exacerbate the strength of the response. Ultimately, the lowered energy available in the host leads to degradation of major cellular structural components which could lead to expulsion of the symbionts from the host tissue. Blue indicates decrease, red indicates an increase. Solid lines indicate processes or pools for which evidence was obtained in this study, stippled lines indicate processes or pools that are hypothesised to change or have been shown to change by Rädecker et al*.* (2021). Arrows indicate positive effect and blocked lines negative effect.

When *in hospite*, the symbiont delivers a major proportion of the reduced energy derived from photosynthesis to the host. Any alteration in this activity and/or in nutrient exchange may be indicative of imminent breakdown of the host-symbiont relationship. In this study, the photosynthetic responses as measured by fluorometry (Fig. 1) were consistent with published studies on bleaching, showing downregulation of photosynthetic efficiency and electron transport in the symbiont under elevated temperatures^7,9,14^. The proteomic data reported here show that declines in photosynthetic efficiency and electron transport coincide with the decreased abundance of proteins associated with photosynthesis, chlorophyll biosynthesis and carbon fixation (Figs. 4 and 5). Congruent with the measured decline in photosynthetic efficiency, we detected a decrease in the abundance of photosynthetic electron transport chain proteins including the cytochrome b6-f complex (-0.68 FC), and the PSII-reaction centre protein subunits, D2 (− 0.56 FC) and D1 (− 0.35 FC). The decline in these PSII subunits are indicative of photoinhibition^14,15,17^, whereby protein repair mechanisms are unable to keep pace with degradation^85^, leading to increased ROS production and subsequent fatty acid oxidation. However, the absence of a strong oxidative stress response in the symbiont suggests a possible controlled downregulation of activity, either to conserve nitrogen use and/or limit ROS production.

Decreases in abundance of several proteins related to photoprotection and photosynthetic pigment biosynthesis were also observed. We detected a strong decrease in Zeaxanthin epoxidase (− 2.75 FC), a protein responsible for converting zeaxanthin into antheraxanthin and PSII stability/assembly factor HCF136 (− 2.43 FC), essential for PSII biogenesis (including psbD and cytochrome b559). Major decreases in abundance were observed in the cases of magnesium protoporphyrin IX methyltransferase (− 3.82 FC) and Mg-chelatase subunit ChlD (− 3.11 FC), proteins involved in chlorophyll biosynthesis, as well as in Glutamate-1-semialdehyde aminotransferase 1, which is involved in the biosynthesis of tetrapyrroles (− 0.55 FC). This predominant decrease in photosynthetic protein complexes and pigment biosynthesis proteins of PSII indicate a possible reduction in PSII units either due to dissociation of light harvesting complexes from the thylakoid membrane because of thermal damage^86,87^ or a decreased dependency on photosynthesis by the symbiont. The increase in peridinin-chlorophyll *a* binding proteins (0.34 FC) however, lends support to the idea that the symbiont is simply altering pigment composition to better suit life outside the symbiosis. Indeed, the significant changes to the symbiont proteome—the decline in photosynthetic performance, absence of extensive oxidative damage, combined with increased accumulation of lipids—might be reflective of the symbiont adjusting to a non-symbiotic lifestyle.

The decrease in light reaction processes was paralleled with a decline in the carbon fixation protein RuBisCO, which decreased -1.46-fold under heat stress. Dinoflagellates contain the bacterial-type (Form II) RuBisCo, which is more thermally stable than the typical plant (Form I) enzyme^88^. Thus, the decreased abundance of RuBisCO may reflect broad changes in symbiont metabolism or DIC limitation from the decreased abundance of V-ATPase (which acts as a CCM).

The results from this study (Fig. 5) are largely consistent with a model recently proposed^22^ in which host energy limitation from reduced carbon translocation results in the collapse of the coral–symbiont relationship. In our study, the increase in proteins involved with proteolysis and lipid catabolism are indicative of energy limitation in the host, and changes in key nitrogen cycling proteins, like those found by Rädecker et al*.* (2021), provide support for possible amino acid breakdown. Additionally, the reduction in symbiont photosynthesis and increased carbon storage provides further evidence for a reduction in carbon translocation to the host, supporting a model of coral symbiosis breakdown based on host energy limitation originating from increased demand and/or reduced carbon flow from the symbionts. Importantly, our study uncovered a decrease in the proton-pump V-ATPase that is known to maintain a low pH in the symbiosome helping concentrate DIC at the host/symbiont interface. This change in symbiosome condition could explain a loss in carbon translocation to the host, as DIC limitation in the symbiont would result in a decline in photosynthesis while also reducing the efficiency of photosynthate translocation through the symbiosome^67^. While the model presented by Rädecker et al*.* 2021, does not attempt to explain symbiont expulsion from the host, our data showed considerable changes to proteins involved in maintaining the structural integrity of the host tissue, and as such, symbionts may be lost through general deterioration of host tissue stability. Finally, our data revealed significant ROS-driven physiological deterioration in the host, which was not as evident in the symbiont. Instead, the symbiont displayed proteomic changes consistent with a general reduction in metabolic activity. While previous studies have established a connection between elevated temperature and ROS production in coral symbionts^70^, the data presented here suggests that, under the environmental conditions employed in this study, the symbionts are able to cope with increased ROS or at least manage it by reducing activity. As such, it is likely that damage in the host is primarily a result of host-produced ROS as opposed to ROS generated by the symbiont, as is frequently proposed^89^.

## Conclusion

**Figure 6:**
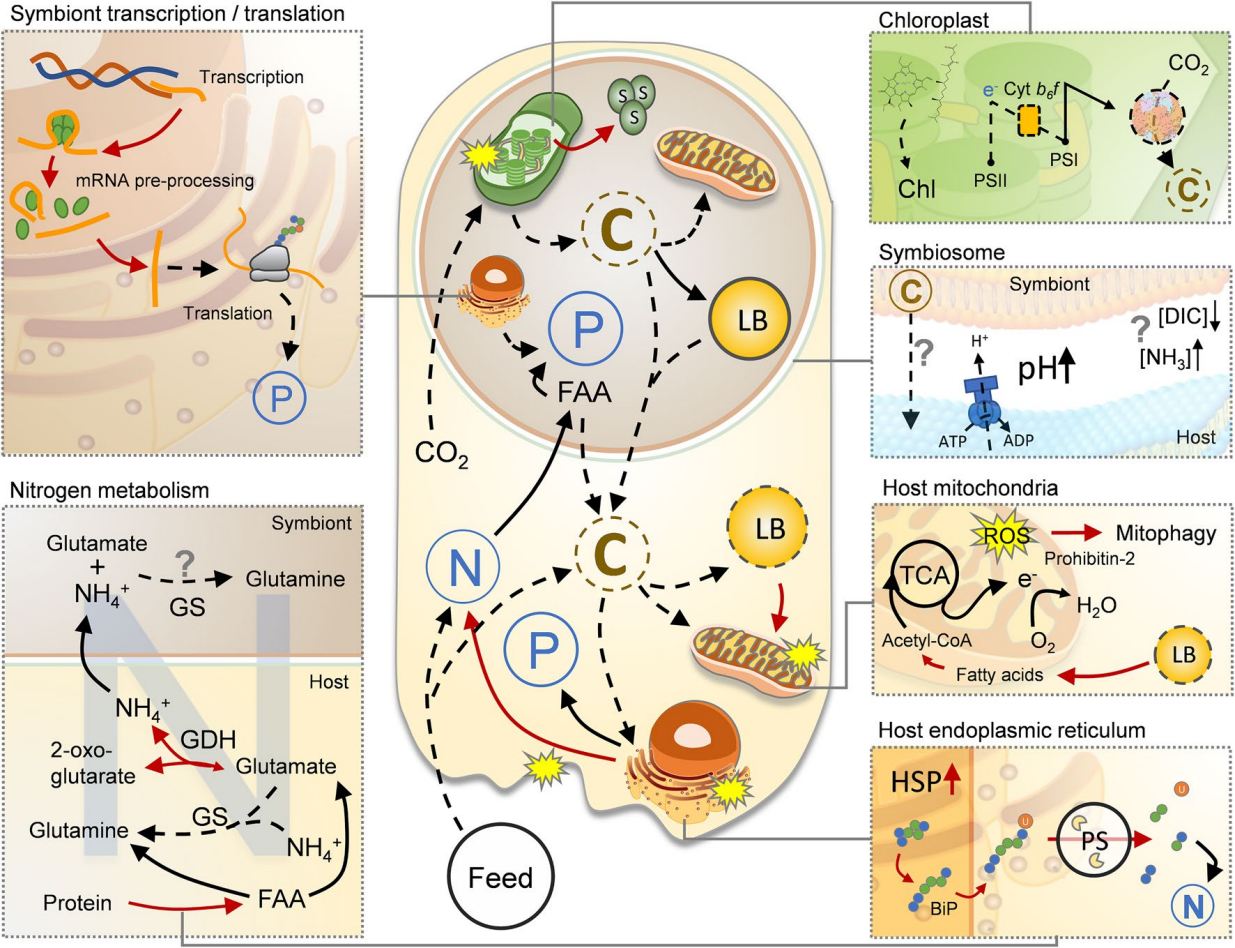
Simplified graphical summary of proposed major physiological changes based on differentially expressed proteins in the host and symbiont of *Acropora millepora* during thermal stress. Broken arrows indicate a decrease and red arrows indicate an increase in protein abundance or pool. Question marks indicate pathways with inconsistent evidence or potential follow-on effects of a change.

Exposure of colonies of *Acropora millepora* to elevated temperatures over six days resulted in loss of endosymbiotic cells from the host tissue as well as a reduction in photosynthetic efficiency. This correlated with major changes in the proteomes of both the host (*Acropora millepora*) and symbiont (*Cladocopium* sp.), implying that the coral colonies were undergoing key metabolic adjustments (Fig. 6). The proteome data indicate that, following the thermal stress regime imposed here, not only were damage limiting proteins (such as HSPs and antioxidants) present at higher levels in the host, but also enzymes related to proteolysis and cell death, suggesting that considerable damage had already occurred in the host. We saw indications that cytoskeletal and tissue structural integrity were compromised in the host, and evidence of impairment to both biomineralization and endoplasmic reticulum function. Importantly, however, no loss or ‘sloughing’ of host tissue was observed at the time of sampling, suggesting the observed changes to tissue integrity had not become systemic. Loss of actin and structural proteins suggest that cell adhesion and attachment processes were impaired, possibly facilitating symbiont loss or expulsion. Altered levels of key proteins involved in nitrogen and fatty acid metabolism provide clear evidence that the host suffered energy limitation under heat stress, presumably a consequence of reduced levels of symbiont photosynthate being translocated. Of significance in the context of bleaching were key changes in proteins that may be direct indicators of shifts in host-symbiont interactions. The decline in host GS (of which high concentrations are associated with symbiosis) and loss of V-ATPase (a key enzyme for acidification of the symbiosome, essential for translocation of metabolites), are likely to directly impair photosynthate translocation and could therefore represent specific indicators of symbiosis breakdown.

In the symbiont, the observed declines in chlorophyll *a* fluorescence and measured physiological changes to photosystem II activity on day six of the heat treatment were mirrored by changes in the symbiont proteome. Declines in levels of photosynthetic and electron transport proteins, as well as many proteins involved in pigment biosynthesis are suggestive of photoinhibition and photodamage, which is often coincident with enhanced ROS production and oxidative stress. However, the symbiont proteome results are inconsistent with the notion of oxidative damage, as no proteins involved in cell death or proteolysis and few antioxidant components were affected by the heat treatment, differing vastly from the host. Metabolic adjustments suggested by the symbiont proteome data include a shift towards glycerol and carbohydrate production, as well as accumulation of storage lipids, seemingly a consequence of diverting fixed carbon away from translocation to the host. The coral-symbiont relationship is a fragile one, and the emerging evidence^22,58^, including the findings of this study, is that its collapse is triggered by disturbances in bilateral nutrient exchange—the details of which may differ between coral and symbiont lineages.

## Methods

### Coral collection and experimental design

Four individual colonies of *Acropora millepora* were collected on the reef flat of Heron Island, Great Barrier Reef, Australia in February 2015. Corals were kept in flow through tanks with 70% shading for 5 days before commencement of thermal stress experiment. Colonies were split in two and one set (*n* = 4) maintained at ambient summer water temperature (control; µ = 28 °C) throughout the experiment (Fig. 1a). Treatment colonies (*n* = 4) assigned to bleaching stress were exposed to temperature increases of 1 °C per day until reaching a maximum of approx. 32 °C (Fig. 1a). A gradual increase in temperature with natural daily fluctuations (night-time cooling) was employed to allow for cellular adjustment in the corals and to avoid degenerative tissue loss from the animal as often occurs during rapid temperature increase. A proxy for coral (photosynthetic) health in situ was obtained by chlorophyll *a* fluorescence using pulse amplitude modulated fluorometry (Mini PAM IMAG-K4, Walz GmbH, Effeltrich, Germany). Measurements were made twice daily, at midday and half an hour after sunset to determine light-adapted effective quantum yield of PSII (ΔF/F_M_') and the dark-adapted maximum quantum yield of PSII (F_V_/F_M_), respectively. Sampling for single-celled fluorometry, macromolecular profiling (FTIR) and proteomics was conducted 48 h after corals reached maximum temperature (31.9 °C) during the onset of bleaching, but before any observed tissue loss.

### Physiological and morphological condition in vivo

Physiological condition of symbiont cells (*in-hospite*) was assessed by single-cell chlorophyll *a* fluorescence and light microscopy. Intact endosymbiotic cells from the genus *Cladocopium* sp. (identified as ITS2 type C8) were extracted from each coral colony following the methods described in Nielsen et al.^70^. The resultant slurry containing a high percentage of intact symbiont cells encased in viable animal cells (*in-hospite*) were gently spun down (500 rpm, ~ 17 RCF), and the pellet subsequently re-suspended in 100 µL FSW and directly analysed for chlorophyll *a* fluorescence or fixed for FTIR microspectroscopy (see below). Variable chlorophyll *a* fluorescence measurements were made on individual *in-hospite* cells (*n* = 80 control; *n* = 80 heated) using a pulse amplitude modulated fluorometer (Imaging PAM IMAG-K4, Walz GmbH, Effeltrich, Germany) mounted on a compound microscope (Axiostar plus, Zeiss, Germany). Measurements were made at 200X magnification employing blue excitation light (440 nm) and collected using the Imaging Win software (V2.32 FW Multi RGB; Walz GmbH, Effeltrich, Germany). After 10 min dark-adaptation, minimum fluorescence (F_O_) was recorded before application of a saturating pulse of light (saturating pulse width = 0.8 s; saturating pulse intensity = 10; using the Special SP-routine), where maximum fluorescence (F_M_) was determined. From these two parameters the quantum yield of PSII was calculated as F_V_/F_M_ = (F_M_-F_O_)/F_M_. To limit the risk of false identification, only easily identifiable host cells including two or more symbionts were measured.

### Coral proteomics, macromolecular composition, cell density and chlorophyll concentration

Coral fragments from each colony were excised and immediately frozen in liquid N_2_ for proteomic analyses or used directly for the determination of symbiont cell density and chlorophyll *a* and *c2* concentrations. Briefly, tissue was released from coral fragments using a high-pressure airgun (water picking) into 5 mL of FSW. For cell density measurements the resulting slurry was homogenised, and symbiont cell concentration determined by manual counting using a haemocytometer (average of 6 counts each). For chlorophyll *a* and *c2* determination the cell slurry was centrifuged at 2000 rpm (~ 2000 RCF) for 5 min and the supernatant discarded. The remaining pellet, consisting primarily of symbiont algal cells, was subsequently resuspended and extracted overnight in 3 mL of chilled 90% acetone, after which the samples were centrifuged again, the chlorophyll *a* and *c*_*2*_ absorption determined spectrophotometrically using wavelengths 630 nm and 664 nm and concentrations calculated according to Ritchie^90^. Cell densities and chlorophyll concentrations were normalised to surface area, which was determined for each coral fragment using a standardised wax technique^91^. For single-cell investigation of macromolecular composition, symbionts (*in-hospite*) were preserved in 2% formalin and stored at room temperature until analysis on the infra-red microspectroscopy beamline at the Australian Synchrotron. Measurements using Fourier Transform Infra-Red (FTIR) spectroscopy were done following the methods of Petrou et al.^10^, see Supplementary Methods for details.

### Protein quantification

Frozen coral fragments were air picked in filtered (0.22 µm) seawater, homogenised on ice and then centrifuged at 4 °C to separate the animal tissue from the algae cells. The animal tissue (supernatant) was immediately snap-frozen in liquid N_2_ and kept at − 80 °C. The algal pellet was re-suspended in filtered seawater and re-centrifuged × 2 to wash away any remnant animal tissue before being pelleted and snap frozen to store at − 80 °C for transport.

Separate samples of animal tissues and symbionts were individually homogenized using a sonicating probe (5 × 30 s), resting tubes between sonication events in a dry ice ethanol bath (30 s). Protein concentrations were measured using the BCA assay microplate kit^92^ and for each sample 100 µg of total protein was isolated for the protein digestion. For details on digestion see Supplementary Methods. Final products were eluted and evaporated to near dryness on a speedvac and reconstituted in 100 µl 2% ACN, 0.1% formic acid and kept at − 80 °C for 2 days prior to being analyzed on the mass spectrometer.

### Mass spectrometry

Duplicate analyses of each peptide sample were completed on the Thermo Scientific Q- Exactive (San Jose, CA) tandem mass spectrometer that was in-line with the Waters nanoAcquity UPLC chromatography system. Peptides were concentrated and then separated using an acidified (0.01% formic acid) acetonitrile: water gradient of 5–35%. Sample analyses were randomized, and quality controls were analyzed every 5^th^ injection. Select peptides from QC samples were monitored using Skyline^93^ to ensure that peptide peak area correlation variances were < 10% through the duration of the analyses. For full details see Supplementary Methods.

### Protein data analysis

Please refer to the Supplementary Methods for full details of protein data analysis. Briefly, raw data files were analysed by Peaks Studio v8.0 (Bioinformatics Solutions, Waterloo, ON) against a database compiled from the translated genomes of the coral host *Acropora millepora*^94^ as well as that of two coral symbionts (*Breviolium minutum,* and *Fugacium kawagutii*) and a database of contaminants. The search results were imported into Scaffold (v4.8.3, Proteome Software Inc., Portland, OR) and used to validate MS/MS based peptide and protein identifications. Only proteins with a minimum of 2 peptide matches, 95% protein threshold and 95% peptide threshold were included for further analyses and exported for data quality validation. Total spectral counts for each protein and sample were normalised to the sum of spectral counts for each sample (16 samples in total, 8 biological samples and two technical replicates for each) and organism. The mean of two technical replicates for each sample and protein was calculated and Levene’s test used to test for equality of variances in mean spectral counts across treatments for each protein. In cases where spectral counts exhibited uneven variance across treatments (P_Leven’s_ < 0.05) the spectral counts were log_10_ transformed prior to test for difference between treatments using a paired t-test (n = 4, *P* < 0.05). To ensure data quality, only proteins where the detected change in abundance could be validated by at least three replicate samples within a given treatment were included for further analysis. Proteins were annotated using the BLAST function in the OmicsBox software (OmicsBox 1.1.78^95^), against the Swissprot database (e-value 10^–5^) or the non-redundant (nr) database (e-value 10^–10^), and subsequently processed through the successive steps of functional annotation provided by the software (GO-term “Mapping”, “InterProScan”, “Merging” of GO-terms and “Annotation” to retain GO-terms with highest scores). In cases where multiple sequences were identified as relating to the same protein (identical name or protein ID) only information for the sequence with the highest total spectral count was retained. Functional information for each protein was extracted from UniProt using the protein ID. Proteins were then categorised into functional groups according to their GO-terms using a custom R-script that allowed for grouping a protein with a given GO-term into any relevant parent (linked higher level) GO-term group. Any proteins with no or limited GO-term information was manually annotated into functional groups using a literature search.

### Statistical analyses

Chlorophyll *a* fluorescence data were analysed for statistical differences over time and between treatments using repeated measures analysis of variance (rmANOVA). Pooled populations of single cell F_V_/F_M_ of *in-hospite* cells from four independent colony fragments were tested for non-equal distributions between control and heated corals using Kolmogorov Smirnov and medians compared for statistical difference. Normalised cell density, chlorophyll data and biomolecular content were analysed for statistical significance using paired T-tests. All data were analysed in R, and differences were considered significant at *P* < 0.05.

## Supplementary Information

Supplementary Information 1.

Supplementary Information 2.

Supplementary Information 3.

Supplementary Information 4.

## Data Availability

All proteomic data generated and analysed during this study are included in this published article (and its supplementary information files). The peptides identified in the current study are available from PRIDE, accession # PXD011668. All other datasets used and/or analysed during the current study are available from the corresponding author on reasonable request.
