# Supplementary material for: Broad scale proteomic analysis of heat-destabilised symbiosis in the hard coral Acropora millepora
Source: Sci Rep. 2021 Sep 24;11:19061. doi: 10.1038/s41598-021-98548-x (PMC8463592; doi:10.1038/s41598-021-98548-x)
Supplement: Supplementary file 2 — Supplementary Information 2. [file 41598_2021_98548_MOESM2_ESM.docx]

Supplementary Table 1. Infrared band assignments of Symbiont cells isolated from the coral *Acropora millepora.*

| Peak & range (cm^-1^) | | | | Assignments | Biological molecules |
| --- | --- | --- | --- | --- | --- |
| 3015 |  | 3020-3006 | olefinic=C-H stretching | | Unsaturated fatty acids^†^ |
| 2964 |  | 2971-2052 | C-H mainly methyl groups from proteins | | Protein (CH-stretch II) ^†^ |
| 2917 |  | 2935-2910 | methylene groups from saturated fatty acids | | Saturated fatty acids^†^ |
| 2850 |  | 2858-2844 |  | | Saturated lipid (CH-stretch IV)^†^ |
| 1745 |  | 1750-1731 | Saturated ester C=O stretching. Ester functional groups primarily from lipids and fatty acids | | Ester carbonyls^‡^ |
| 1544 |  | 1560-1533 | N-H of amides associated with proteins | | Amide II^‡^ |
| 1475 |  | 1484-1465 |  | | Free amino acids I |
| 1419 |  | 1425-1413 |  | | Free amino acids II |
| 1375 |  | 1400-1363 | COO^-^ | | Carboxylates |
| 1264 |  | 1270-1255 | P=O of the phosphodiester backbone of nucleic acid or presence of phosphorylated proteins | | Phosphorylated compounds^‡^ |
| 1174 |  | 1188-1164 | C-O-C stretching | | Carbohydrates I^‡^ |
| 1154 |  | 1162-1140 |  | | Carbohydrates II^‡^ |

^†^Vongsvivut *et al*., (2012)
^‡^Giordano *et al*., (2001)
