# Supplementary material for: Broad scale proteomic analysis of heat-destabilised symbiosis in the hard coral Acropora millepora
Source: Sci Rep. 2021 Sep 24;11:19061. doi: 10.1038/s41598-021-98548-x (PMC8463592; doi:10.1038/s41598-021-98548-x)
Supplement: Supplementary file 3 — Supplementary Information 3. [file 41598_2021_98548_MOESM3_ESM.docx]

Supplementary Table 2. Differentially expressed proteins in the host of *Acropora millepora*

| protein_id | protein | e_value | log2_change | p_value | hange | functional groups |
| --- | --- | --- | --- | --- | --- | --- |
| Q99K85 | Phosphoserine aminotransferase (PSAT) (EC 2.6.1.52) (Endometrial progesterone-induced protein) (EPIP) (Phosphohydroxythreonine aminotransferase) | 1.58E-163 | -3.63 | 0.0341 | down | Nitrogen metabolism [GO] |
| XP0291884311 | serine protease | 0 | -3.57 | 0.0433 | down | Protein degradation [manual] |
| B3EWY7 | Acidic skeletal organic matrix protein (Acidic SOMP) | 0 | -2.3 | 0.0209 | down | Extracellular matrix [manual] |
| P61354 | 60S ribosomal protein L27 | 7.83E-70 | -2.13 | 0.0488 | down | Translation [GO] |
| Q99NB1 | Acetyl-coenzyme A synthetase 2-like, mitochondrial (EC 6.2.1.1) (Acetate--CoA ligase 2) (Acetyl-CoA synthetase 2) (AceCS2) (Acyl-CoA synthetase short-chain family member 1) (Propionate--CoA ligase) (EC 6.2.1.17) | 0 | -2.01 | 0.038 | down | Fatty acid and lipid metabolism [manual] |
| XP0157672031 | PREDICTED: uncharacterized protein LOC107345966 isoform X3 | 0 | -1.86 | 0.0411 | down | Other [manual] |
| P15104 | Glutamine synthetase (GS) (EC 6.3.1.2) (Glutamate--ammonia ligase) (Palmitoyltransferase GLUL) (EC 2.3.1.225) | 2.48E-152 | -1.55 | 2.00E-04 | down | Nitrogen metabolism [GO] |
| Q9Y5Z4 | Heme-binding protein 2 (Placental protein 23) (PP23) (Protein SOUL) | 4.15E-31 | -1.55 | 4.00E-04 | down | Cell death [GO], Response to stress [manual] |
| P00918 | Carbonic anhydrase 2 (EC 4.2.1.1) (Carbonate dehydratase II) (Carbonic anhydrase C) (CAC) (Carbonic anhydrase II) (CA-II) | 1.48E-77 | -1.2 | 0.0309 | down | Carbon fixation [GO] |
| P19615 | Major yolk protein (MYP) (Vitellogenin) | 1.74E-72 | -1.16 | 0.0487 | down | Other [manual] |
| O75525 | KH domain-containing, RNA-binding, signal transduction-associated protein 3 (RNA-binding protein T-Star) (Sam68-like mammalian protein 2) (SLM-2) (Sam68-like phosphotyrosine protein) | 4.77E-41 | -1.14 | 0.0134 | down | Other [manual] |
| P26007 | Integrin alpha-6 (VLA-6) [Cleaved into: Integrin alpha-6 heavy chain; Integrin alpha-6 light chain] | 1.26E-84 | -1.06 | 0.0422 | down | Extracellular matrix [GO] |
| Q6GLT8 | Fumarylacetoacetate hydrolase domain-containing protein 2 (EC 3.-.-.-) | 4.90E-144 | -0.93 | 0.0303 | down | Nitrogen metabolism [manual] |
| Q07130 | UTP--glucose-1-phosphate uridylyltransferase (EC 2.7.7.9) (UDP-glucose pyrophosphorylase) (UDPGP) (UGPase) | 0 | -0.91 | 0.0109 | down | Carbohydrate metabolism [manual] |
| P00920 | Carbonic anhydrase 2 (EC 4.2.1.1) (Carbonate dehydratase II) (Carbonic anhydrase II) (CA-II) | 4.86E-51 | -0.87 | 0.032 | down | Carbon fixation [GO] |
| O97490 | Melanotransferrin (Membrane-bound transferrin-like protein p97) (CD antigen CD228) | 1.06E-69 | -0.85 | 0.0267 | down | Protein modification [GO], Signal transduction [GO] |
| P13676 | Acylamino-acid-releasing enzyme (AARE) (EC 3.4.19.1) (Acyl-peptide hydrolase) (APH) (Acylaminoacyl-peptidase) | 2.79E-118 | -0.83 | 0.0203 | down | Protein degradation [GO] |
| O00468 | Agrin [Cleaved into: Agrin N-terminal 110 kDa subunit; Agrin C-terminal 110 kDa subunit; Agrin C-terminal 90 kDa fragment (C90); Agrin C-terminal 22 kDa fragment (C22)] | 4.76E-50 | -0.79 | 0.0083 | down | Other [manual] |
| Q2HJ57 | Coactosin-like protein | 5.13E-39 | -0.79 | 0.0298 | down | Cell structure and organisation [GO] |
| P60901 | Proteasome subunit alpha type-6 (EC 3.4.25.1) (Macropain iota chain) (Multicatalytic endopeptidase complex iota chain) (Proteasome iota chain) | 2.80E-152 | -0.71 | 0.0118 | down | Protein degradation [GO] |
| Q60631 | Growth factor receptor-bound protein 2 (Adapter protein GRB2) (SH2/SH3 adapter GRB2) | 1.09E-95 | -0.65 | 0.0415 | down | Signal transduction [manual] |
| P78417 | Glutathione S-transferase omega-1 (GSTO-1) (EC 2.5.1.18) (Glutathione S-transferase omega 1-1) (GSTO 1-1) (Glutathione-dependent dehydroascorbate reductase) (EC 1.8.5.1) (Monomethylarsonic acid reductase) (MMA(V) reductase) (EC 1.20.4.2) (S-(Phenacyl)glutathione reductase) (SPG-R) | 2.84E-54 | -0.62 | 0.0152 | down | Antioxidants [manual] |
| Q761X5 | Netrin receptor UNC5C (Protein unc-5 homolog 3) (Protein unc-5 homolog C) | 4.81E-07 | -0.61 | 0.0195 | down | Cell death [manual] |
| Q5TTG1 | V-type proton ATPase catalytic subunit A (V-ATPase subunit A) (EC 7.1.2.2) (V-ATPase 69 kDa subunit) (Vacuolar proton pump subunit alpha) | 0 | -0.6 | 0.0162 | down | Transmembrane transport [GO] |
| P48758 | Carbonyl reductase [NADPH] 1 (EC 1.1.1.184) (15-hydroxyprostaglandin dehydrogenase [NADP(+)]) (EC 1.1.1.197) (NADPH-dependent carbonyl reductase 1) (Prostaglandin 9-ketoreductase) (Prostaglandin-E(2) 9-reductase) (EC 1.1.1.189) | 1.57E-117 | -0.59 | 0.0274 | down | Fatty acid and lipid metabolism [GO] |
| Q5XIT1 | Microtubule-associated protein RP/EB family member 3 (EB1 protein family member 3) (EBF3) (End-binding protein 3) (EB3) (RP3) | 1.05E-99 | -0.54 | 0.0195 | down | Cell structure and organisation [GO] |
| Q91WV7 | Neutral and basic amino acid transport protein rBAT (Solute carrier family 3 member 1) (b(0,+)-type amino acid transport protein) (NBAT) | 4.88E-105 | -0.48 | 0.0272 | down | Carbohydrate metabolism [GO] |
| Q14247 | Src substrate cortactin (Amplaxin) (Oncogene EMS1) | 1.66E-173 | -0.45 | 0.0205 | down | Cell structure and organisation [manual], Intracellular transport [manual] |
| Q8WWF8 | Calcyphosin-like protein | 2.62E-44 | -0.44 | 0.0296 | down | Other [manual] |
| Q862Z5 | Cystatin-B (Stefin-B) | 1.63E-31 | -0.43 | 0.0088 | down | Protein degradation [manual] |
| Q925G0 | RNA-binding protein 3 (RNA-binding motif protein 3) | 1.85E-18 | -0.43 | 0.0254 | down | Response to stress [manual], Translation [manual] |
| P72745 | Universal stress protein Slr1101 (USP Slr1101) | 2.38E-11 | -0.36 | 0.0196 | down | Response to stress [manual] |
| P69526 | Transmembrane protease serine 9 (EC 3.4.21.-) (Polyserase-I) (Polyserine protease 1) (Polyserase-1) [Cleaved into: Serase-1; Serase-2; Serase-3] | 4.24E-73 | -0.34 | 0.0012 | down | Protein degradation [GO] |
| B1H120 | Septin-8 | 0 | -0.29 | 0.05 | down | Cell structure and organisation [manual] |
| Q5ZJF4 | Peroxiredoxin-6 (EC 1.11.1.15) (1-Cys peroxiredoxin) (1-Cys PRX) (Acidic calcium-independent phospholipase A2) (aiPLA2) (EC 3.1.1.4) (Non-selenium glutathione peroxidase) (NSGPx) | 1.39E-110 | -0.28 | 0.019 | down | Antioxidants [GO] |
| Q6QEF8 | Coronin-6 (Coronin-like protein E) (Clipin-E) | 5.88E-149 | -0.27 | 0.0097 | down | Intercellular transport [manual] |
| Q6PH57 | Guanine nucleotide-binding protein G(I)/G(S)/G(T) subunit beta-1 (Transducin beta chain 1) | 0 | -0.23 | 0.003 | down | Signal transduction [GO] |
| Q9BGI1 | Peroxiredoxin-5, mitochondrial (EC 1.11.1.15) (Peroxiredoxin V) (Prx-V) (Thioredoxin peroxidase) | 2.56E-43 | -0.23 | 0.0149 | down | Antioxidants [manual] |
| P19804 | Nucleoside diphosphate kinase B (NDK B) (NDP kinase B) (EC 2.7.4.6) (Histidine protein kinase NDKB) (EC 2.7.13.3) (P18) | 6.66E-82 | -0.22 | 0.0097 | down | Other [manual] |
| Q5R7G6 | Probable aminopeptidase NPEPL1 (EC 3.4.11.-) (Aminopeptidase-like 1) | 0 | -0.22 | 0.0185 | down | Protein degradation [GO] |
| P54985 | Peptidyl-prolyl cis-trans isomerase (PPIase) (EC 5.2.1.8) (Cyclophilin) (Cyclosporin A-binding protein) (Rotamase) | 3.03E-92 | -0.2 | 0.0298 | down | Protein modification [GO] |
| K0J107 | Malate dehydrogenase, mitochondrial (EC 1.1.1.37) | 6.32E-96 | -0.19 | 0.0052 | down | Protein modification [GO], Carbohydrate metabolism [GO] |
| P25324 | Thiosulfate sulfurtransferase (EC 2.8.1.1) (Rhodanese) | 2.60E-61 | -0.19 | 0.0361 | down | Sulfur metabolism [GO] |
| Q05187 | Hemocyte protein-glutamine gamma-glutamyltransferase (EC 2.3.2.13) (Hemocyte transglutaminase) (TGase) | 1.31E-100 | -0.18 | 0.0383 | down | Protein modification [GO] |
| Q63028 | Alpha-adducin (Erythrocyte adducin subunit alpha) | 5.24E-109 | -0.15 | 0.0112 | down | Response to stress [GO], Cell structure and organisation [GO], Signal transduction [GO], Transmembrane transport [GO] |
| Q8BTM8 | Filamin-A (FLN-A) (Actin-binding protein 280) (ABP-280) (Alpha-filamin) (Endothelial actin-binding protein) (Filamin-1) (Non-muscle filamin) | 0 | -0.14 | 0.0076 | down | Cell structure and organisation [manual] |
| Q15334 | Lethal(2) giant larvae protein homolog 1 (LLGL) (DLG4) (Hugl-1) (Human homolog to the D-lgl gene protein) | 1.48E-126 | -0.12 | 0.0375 | down | Cell structure and organisation [manual] |
| P35241 | Radixin | 0 | -0.08 | 0.0236 | down | Cell structure and organisation [GO] |
| P42700 | 60 kDa SS-A/Ro ribonucleoprotein (60 kDa Ro protein) (60 kDa ribonucleoprotein Ro) (Ro60) (RoRNP) (TROVE domain family member 2) | 0 | -Inf | 0 | down | Response to stress [manual] |
| O95490 | Adhesion G protein-coupled receptor L2 (Calcium-independent alpha-latrotoxin receptor 2) (CIRL-2) (Latrophilin homolog 1) (Latrophilin-2) (Lectomedin-1) | 2.41E-43 | -Inf | 0 | down | Signal transduction [GO] |
| XP0292085191 | polyadenylate-binding protein-interacting protein 1-like isoform X1 | 6.33E-153 | -Inf | 0 | down | Translation [manual] |
| XP0157499721 | PREDICTED: uncharacterized protein LOC107329816 | 1.11E-58 | -Inf | 0 | down | Protein degradation [GO] |
| XP0291924981 | uncharacterized protein LOC114958819 | 0 | -Inf | 0 | down | Other [manual] |
|  |  |  |  |  |  |  |
| Q9CQ52 | Chymotrypsin-like elastase family member 3B (EC 3.4.21.70) (Elastase IIIB) (Elastase-3B) (Protease E) | 1.89E-28 | 0.09 | 0.0285 | up | Protein degradation [GO] |
| Q5RDG4 | Protein disulfide-isomerase A3 (EC 5.3.4.1) | 4.54E-151 | 0.21 | 0.0315 | up | Protein modification [manual] |
| P55214 | Caspase-7 (CASP-7) (EC 3.4.22.60) (Apoptotic protease Mch-3) (ICE-like apoptotic protease 3) (ICE-LAP3) (SREBP cleavage activity 2) (SCA-2) [Cleaved into: Caspase-7 subunit p20; Caspase-7 subunit p11] | 1.10E-66 | 0.23 | 0.0315 | up | Cell death [GO], Protein degradation [GO] |
| P34737 | 40S ribosomal protein S15 (S12) | 2.23E-81 | 0.26 | 0.0177 | up | Translation [GO] |
| P82471 | Guanine nucleotide-binding protein G(q) subunit alpha (Guanine nucleotide-binding protein alpha-q) | 0 | 0.31 | 0.0303 | up | Signal transduction [GO] |
| P14314 | Glucosidase 2 subunit beta (80K-H protein) (Glucosidase II subunit beta) (Protein kinase C substrate 60.1 kDa protein heavy chain) (PKCSH) | 7.64E-128 | 0.41 | 0.0494 | up | Protein modification [manual] |
| Q9JJW6 | Aly/REF export factor 2 (Alyref) (RNA and export factor-binding protein 2) | 3.98E-64 | 0.43 | 0.0084 | up | Transcription [manual] |
| Q9JI85 | Nucleobindin-2 (DNA-binding protein NEFA) (Prepronesfatin) [Cleaved into: Nesfatin-1] | 6.08E-80 | 0.43 | 0.0301 | up | Other [manual] |
| P08003 | Protein disulfide-isomerase A4 (EC 5.3.4.1) (Endoplasmic reticulum resident protein 72) (ER protein 72) (ERp-72) (ERp72) | 0 | 0.45 | 4.00E-04 | up | Protein modification [manual] |
| Q98SJ2 | DAZ-associated protein 1 (Deleted in azoospermia-associated protein 1) (Proline-rich Vg1 mRNA-binding protein) | 9.09E-48 | 0.48 | 0.0443 | up | Other [manual] |
| P30946 | Heat shock protein HSP 90-alpha | 5.58E-127 | 0.48 | 0.0141 | up | Protein modification [GO] |
| O08807 | Peroxiredoxin-4 (EC 1.11.1.15) (Antioxidant enzyme AOE372) (Peroxiredoxin IV) (Prx-IV) (Thioredoxin peroxidase AO372) (Thioredoxin-dependent peroxide reductase A0372) | 9.04E-134 | 0.48 | 0.0235 | up | Antioxidants [GO] |
| Q9K9H0 | Isocitrate lyase (ICL) (EC 4.1.3.1) (Isocitrase) (Isocitratase) | 0 | 0.5 | 0.0484 | up | Carbohydrate metabolism [GO] |
| Q2TBQ5 | 60S ribosomal protein L7a | 6.35E-124 | 0.52 | 0.0042 | up | mRNA metabolic process [GO], Translation [GO], Nitrogen metabolism [GO], Intracellular transport [GO], Nitrogen transport [GO] |
| P10860 | Glutamate dehydrogenase 1, mitochondrial (GDH 1) (EC 1.4.1.3) (Memory-related gene 2 protein) (MRG-2) | 0 | 0.54 | 0.0396 | up | Nitrogen metabolism [manual] |
| Q5U2V4 | Phospholipase B-like 1 (EC 3.1.1.-) (LAMA-like protein 1) (Lamina ancestor homolog 1) (Phospholipase B domain-containing protein 1) [Cleaved into: Phospholipase B-like 1 chain A; Phospholipase B-like 1 chain B; Phospholipase B-like 1 chain C] | 2.93E-149 | 0.54 | 0.0413 | up | Fatty acid and lipid metabolism [manual] |
| P79403 | Neutral alpha-glucosidase AB (EC 3.2.1.207) (Alpha-glucosidase 2) (Glucosidase II subunit alpha) | 0 | 0.59 | 0.0439 | up | Carbohydrate metabolism [GO] |
| Q66HD0 | Endoplasmin (94 kDa glucose-regulated protein) (GRP-94) (Heat shock protein 90 kDa beta member 1) | 1.37E-166 | 0.65 | 0.0422 | up | Protein modification [GO] |
| P61314 | 60S ribosomal protein L15 | 2.29E-117 | 0.66 | 0.0376 | up | Translation [GO] |
| Q9Z1Q9 | Valine--tRNA ligase (EC 6.1.1.9) (Protein G7a) (Valyl-tRNA synthetase) (ValRS) | 0 | 0.83 | 0.0397 | up | Translation [GO] |
| Q90593 | Endoplasmic reticulum chaperone BiP (EC 3.6.4.10) (78 kDa glucose-regulated protein) (GRP-78) (Binding-immunoglobulin protein) (BiP) (Heat shock protein 70 family protein 5) (HSP70 family protein 5) (Heat shock protein family A member 5) (Immunoglobulin heavy chain-binding protein) | 0 | 0.84 | 0.0254 | up | Cell death [GO], Protein degradation [GO], Protein modification [GO], Response to stress [GO], Signal transduction [GO], Intracellular transport [GO], Nitrogen transport [GO], Transmembrane transport [GO] |
| Q5VZK9 | F-actin-uncapping protein LRRC16A (CARMIL homolog) (Capping protein regulator and myosin 1 linker protein 1) (Capping protein, Arp2/3 and myosin-I linker homolog 1) (Capping protein, Arp2/3 and myosin-I linker protein 1) (Leucine-rich repeat-containing protein 16A) | 0 | 0.93 | 0.034 | up | Cell structure and organisation [manual] |
| Q8CJ40 | Rootletin (Ciliary rootlet coiled-coil protein) | 0 | 0.96 | 0.034 | up | Cell structure and organisation [GO] |
| Q04832 | DNA-binding protein HEXBP (Hexamer-binding protein) | 4.07E-46 | 1.05 | 0.0375 | up | Other [manual] |
| Q9JMH4 | Collagen alpha-1(XVII) chain (180 kDa bullous pemphigoid antigen 2) (Bullous pemphigoid antigen 2) [Cleaved into: 120 kDa linear IgA disease antigen homolog] | 1.10E-13 | 1.08 | 0.0102 | up | Extracellular matrix [manual] |
| Q9Z1G4 | V-type proton ATPase 116 kDa subunit a isoform 1 (V-ATPase 116 kDa isoform a1) (Clathrin-coated vesicle/synaptic vesicle proton pump 116 kDa subunit) (Vacuolar adenosine triphosphatase subunit Ac116) (Vacuolar proton pump subunit 1) (Vacuolar proton translocating ATPase 116 kDa subunit a isoform 1) | 0 | 1.2 | 0.0029 | up | Transmembrane transport [GO] |
| P13804 | Electron transfer flavoprotein subunit alpha, mitochondrial (Alpha-ETF) | 6.34E-164 | 1.29 | 0.0494 | up | Respiration [manual], Fatty acid and lipid metabolism [manual], Nitrogen metabolism [manual] |
| Q13591 | Semaphorin-5A (Semaphorin-F) (Sema F) | 3.95E-12 | 1.34 | 0.007 | up | Cell death [GO], Protein degradation [GO], Response to stress [GO], Intracellular transport [GO] |
| Q16982 | Neurocalcin | 1.29E-113 | 1.46 | 0.0108 | up | Other [manual] |
| Q09109 | Tryptophan 2-monooxygenase (EC 1.13.12.3) | 9.05E-06 | 1.47 | 0.0072 | up | Nitrogen metabolism [manual] |
| Q5XIH7 | Prohibitin-2 (B-cell receptor-associated protein BAP37) (BAP-37) | 1.82E-123 | 1.52 | 0.0177 | up | Transcription [manual], Respiration [manual] |
| Q9V3H2 | 26S proteasome non-ATPase regulatory subunit 14 (EC 3.4.19.-) (26S proteasome regulatory complex subunit p37B) (26S proteasome regulatory subunit rpn11) (Yippee-interacting protein 5) | 6.83E-171 | 1.55 | 0.0266 | up | Protein degradation [GO], Protein modification [GO] |
| XP0291875881 | uncharacterized protein LOC114954993 | 8.52E-114 | 1.65 | 0.0052 | up | Other [manual] |
| XP0291893411 | sporulation-specific protein 15-like | 0 | 1.73 | 0.0464 | up | Other [manual] |
| Q8C0L0 | Thioredoxin-related transmembrane protein 4 (Thioredoxin domain-containing protein 13) | 9.63E-58 | 2.42 | 0.0035 | up | Other [manual] |
| P0C6B8 | Sushi, von Willebrand factor type A, EGF and pentraxin domain-containing protein 1 | 0 | 3.65 | 0.0321 | up | Other [manual] |
| O95433 | Activator of 90 kDa heat shock protein ATPase homolog 1 (AHA1) (p38) | 3.15E-110 | Inf | 0 | up | Protein modification [manual] |
| O43747 | AP-1 complex subunit gamma-1 (Adaptor protein complex AP-1 subunit gamma-1) (Adaptor-related protein complex 1 subunit gamma-1) (Clathrin assembly protein complex 1 gamma-1 large chain) (Gamma1-adaptin) (Golgi adaptor HA1/AP1 adaptin subunit gamma-1) | 0 | Inf | 0 | up | Intracellular transport [GO], Nitrogen transport [GO] |
| P31335 | Bifunctional purine biosynthesis protein PURH [Includes: Phosphoribosylaminoimidazolecarboxamide formyltransferase (EC 2.1.2.3) (5-aminoimidazole-4-carboxamide ribonucleotide formyltransferase) (AICAR transformylase); IMP cyclohydrolase (EC 3.5.4.10) (ATIC) (IMP synthase) (Inosinicase)] | 0 | Inf | 0 | up | Nitrogen metabolism [manual] |
| Q00168 | Calcium/calmodulin-dependent protein kinase type II alpha chain (CaM-kinase II alpha chain) (EC 2.7.11.17) | 0 | Inf | 0 | up | Protein modification [GO] |
| B3EWZ3 | Coadhesin (Fragment) | 2.81E-74 | Inf | 0 | up | Protein degradation [GO] |
| A7SK48 | Eukaryotic translation initiation factor 3 subunit A (eIF3a) (Eukaryotic translation initiation factor 3 subunit 10) | 0 | Inf | 0 | up | Translation [manual] |
| Q9Y2Q3 | Glutathione S-transferase kappa 1 (EC 2.5.1.18) (GST 13-13) (GST class-kappa) (GSTK1-1) (hGSTK1) (Glutathione S-transferase subunit 13) | 3.44E-82 | Inf | 0 | up | Antioxidants [manual] |
| Q7ZTY4 | Histone-binding protein RBBP7 (Retinoblastoma-binding protein 7) (RBBP-7) | 0 | Inf | 0 | up | DNA maintenance [manual], Transcription [manual] |
| O08746 | Matrilin-2 | 2.10E-52 | Inf | 0 | up | Extracellular matrix [manual] |
| Q5XIF3 | NADH dehydrogenase [ubiquinone] iron-sulfur protein 4, mitochondrial (Complex I-18 kDa) (CI-18 kDa) (NADH-ubiquinone oxidoreductase 18 kDa subunit) | 8.73E-56 | Inf | 0 | up | Respiration [manual] |
| O00567 | Nucleolar protein 56 (Nucleolar protein 5A) | 0 | Inf | 0 | up | Translation [manual] |
| O22943 | Probable glycosyltransferase STELLO1 (EC 2.4.-.-) | 8.05E-95 | Inf | 0 | up | Intracellular transport [manual] |
| Q9SUR9 | Protein SGT1 homolog A (AtSGT1a) (Suppressor of G2 allele of SKP1 homolog A) | 1.98E-75 | Inf | 0 | up | Protein modification [manual] |
| Q91X83 | S-adenosylmethionine synthase isoform type-1 (AdoMet synthase 1) (EC 2.5.1.6) (Methionine adenosyltransferase 1) (MAT 1) | 3.13E-180 | Inf | 0 | up | Sulfur metabolism [GO], Nitrogen metabolism [manual] |
| Q2VLG4 | Scavenger receptor cysteine-rich type 1 protein M130 (CD antigen CD163) [Cleaved into: Soluble CD163 (sCD163)] | 1.40E-86 | Inf | 0 | up | Intracellular transport [GO] |
| P01251 | Splenin (Thymopoietin III) | 2.58E-11 | Inf | 0 | up | Other [manual] |
| Q5REU4 | Staphylococcal nuclease domain-containing protein 1 (EC 3.1.31.1) (100 kDa coactivator) (p100 co-activator) | 0 | Inf | 0 | up | Nitrogen metabolism [GO] |
| Q9Y6N5 | Sulfide:quinone oxidoreductase, mitochondrial (SQOR) (EC 1.8.5.-) (Sulfide dehydrogenase-like) (Sulfide quinone oxidoreductase) | 7.72E-67 | Inf | 0 | up | Sulfur metabolism [manual] |
| O57382 | Tolloid-like protein 2 (EC 3.4.24.-) (Metalloprotease xolloid) (Xenopus tolloid) | 8.69E-41 | Inf | 0 | up | Protein degradation [manual] |
| Q5R4X4 | Translocon-associated protein subunit alpha (TRAP-alpha) (Signal sequence receptor subunit alpha) (SSR-alpha) | 1.74E-78 | Inf | 0 | up | Transmembrane transport [manual] |
| Q8R1V4 | Transmembrane emp24 domain-containing protein 4 (Endoplasmic reticulum stress-response protein 25) (ERS25) (p24 family protein alpha-3) (p24alpha3) (p26) | 5.19E-120 | Inf | 0 | up | Intracellular transport [GO], Nitrogen transport [GO] |
| XP0291913031 | uncharacterized protein LOC114957897 | 0 | Inf | 0 | up | Other [manual] |
| Q24JV4 | UPF0390 protein zgc136864 | 5.98E-11 | Inf | 0 | up | Other [manual] |
| Q5ZIL2 | Vacuolar protein sorting-associated protein 29 (Vesicle protein sorting 29) | 6.29E-112 | Inf | 0 | up | Intracellular transport [GO] |
