# Supplementary material for: Broad scale proteomic analysis of heat-destabilised symbiosis in the hard coral Acropora millepora
Source: Sci Rep. 2021 Sep 24;11:19061. doi: 10.1038/s41598-021-98548-x (PMC8463592; doi:10.1038/s41598-021-98548-x)
Supplement: Supplementary file 4 — Supplementary Information 4. [file 41598_2021_98548_MOESM4_ESM.docx]

SUPPLEMENTARY TEXT FILE

Broad scale proteomic analysis of heat-destabilised symbiosis in the hard coral *Acropora millepora*

Petrou K^1#*^, Nunn BL^2^, Padula MP^1^, David Miller^3^, Nielsen DA^1#^

Contains:

Supplementary Methods

Supplementary Results and Discussion

**Supplementary Methods**

*Synchrotron-based FTIR microspectroscopy*

Measurements were made on hydrated formalin fixed *in-hospite* cells, and spectra acquired over the measurement range 4000-800 cm^-1^ with a Vertex 80v Fourier Transform Infra-Red (FTIR) spectrometer (Bruker Optics, Ettlingen, Germany) following the methods of Petrou et al. ^1^. Spectral measurements (64 co-added scans) were made on single, double and triple endosymbiotic cells using transmission mode (aperture size 4 µm x 4µm). Number of cells measured for each treatment (n = 20-30) varied depending on density of sample and thus ease of locating cells. Multiple spectra (3-5) from each cell were averaged and exported for analysis using R ^2^. The regions that contained the major biological bands were selected (Supplementary Table 1), data were smoothed (4pts either side) and second derivative (3^rd^ polynomial) transformed using Savitzky-Golay algorithm from the prospectr package ^3^ and then normalised using the method of Single Normal Variate (SNV). Macromolecular content for individual species was estimated based on integrating the area under each assigned peak, providing a relative approximation of metabolite content according to the Beer - Lambert Law, which assumes a direct relationship between absorbance and analyte concentration ^4^.

*Protein digestion*

Each digested protein sample was resuspended to a final volume of 100 µl 6M urea in 50 mM ammonium bicarbonate. Samples received 1.5 M Tris pH 8.8 and were reduced with 2.5 µl 200 mM TCEP (1 hour at 37 °C). Samples were cooled and proteins were alkylated with 20 µl of 200 mM iodoacetamide (IAA; 1 hour, 20 °C, in the dark). Then, each sample was reduced again with 20 µl 200 mM diothiothreitol (DTT; 1 hour, 20 °C) prior to receiving the first enzyme treatment, LysC, at a 1:20 enzyme: protein ratio (1 hr, 20 °C). LysC cleaves at C-term of R and K, like trypsin, but works well in 6M urea solutions, allowing more complete digestion of membrane-bound proteins. Each sample then received 800 µl 25 mM NH _4_HCO_3_ and 200 µl HPLC grade methanol and 5 µg trypsin (Pierce^™^ Trypsin Protease MS-Grade; 12 hr, 20 °C). Samples were then evaporated to near dryness on speed vacuum (4 °C) and resuspended in 200 µl 5% acetonitrile + 0.1% trifluoroacetic acid and the finally, pH was adjusted to <2. Samples were desalted using Nest group Micro-Spin columns following the manufacturer’s instructions. Peptides were eluted and evaporated to near dryness on a speedvac and reconstituted in 100 µl 2% ACN, 0.1% formic acid and kept at -80 °C for 2 days prior to being analyzed on the mass spectrometer.

*Mass Spectrometry*

Duplicate analyses of each peptide sample were completed on the Thermo Scientific Q- Exactive (San Jose, CA) tandem mass spectrometer that was in-line with the Waters nanoAcquity UPLC chromatography system. Peptides were concentrated on a 3 cm pre-column (150 µm inner diameter) at 200 nl min^-1^ for 10 min, and separated using a 30 cm analytical column (100 µm inner diameter); both columns were packed with 3 µm C18 beads (ReproSil-Pur C18AQ; Dr. Maisch Ammerbusch Germany**).**  Peptides were separated using an acidified (0.01% formic acid) acetonitrile: water gradient of 5–35% over 90 min with a flow rate of 250 nl min^-1^. MS1 data was collected on 400-1400 m/z with a 70,000 resolution and AGC target of 1e6, while the MS2 data were collected with a loop count of 20 excluding +1 and >+6 MS1 ions (10s dynamic exclusion), 35,000 resolution, and AGC target of 5e^4^.  Sample analyses were randomized, and quality controls were analyzed every 5^th^ injection.  Select peptides from QC samples were monitored using Skyline ^5^ to ensure that peptide peak area correlation variances were <10% through the duration of the analyses.

*Protein data analysis*

Raw data files were analysed by Peaks Studio v8.0 (Bioinformatics Solutions, Waterloo, ON) against a database compiled from the translated genomes of the coral host *Acropora millepora* ^6^ as well as that of the symbiont. At the time of data processing, only two incomplete genomes had been published of coral symbionts *S. minutum* ^7^ and *S. kawagutii* ^8^, now *Breviolium minutum* and *Fugacium kawagutii*, respectively ^9^. Therefore, to increase coverage of the symbiont proteome, the symbiont peptide data was run against both genome-predicted proteomes and a database of common contaminants (63,665 sequences in total; see further information below) with the following parameter settings. Fixed modifications: none. Variable modifications: carbamidomethyl, oxidised methionine, deamidated asparagine and glutamine. Enzyme: semi-trypsin. Number of allowed missed cleavages: 3. Peptide mass tolerance: 20 ppm. MS/MS mass tolerance: 0.1 Da. The results of the search were then filtered to include peptides with a –log_10_P score that was determined by the False Discovery Rate (FDR) of <1%, the score being that where decoy database search matches were <1% of the total matches. Search results were then exported as mzXML files for further analysis with Scaffold.

The search results as mzXML format were imported into Scaffold (v4.8.3, Proteome Software Inc., Portland, OR) and used to validate MS/MS based peptide and protein identifications. Peptide identifications were accepted if they could be established at greater than 80.0 % probability by the Peptide Prophet algorithm ^10^ with Scaffold delta-mass correction and Peptide FDR was 0.7 %, while protein identifications were accepted if they could be established at greater than 80.0 % probability and contained at least 1 identified peptide. Protein probabilities were assigned by the Protein Prophet algorithm ^11^ at a FDR of 1.5%. Proteins that contained similar peptides and could not be differentiated based on MS/MS analysis alone were grouped to satisfy the principles of parsimony. Proteins sharing significant peptide evidence were grouped into clusters. Only proteins with a minimum of 2 peptide matches, 95% protein threshold and 95% peptide threshold were included for further analyses, and raw spectral counts per validated protein, sample, replicate and organism were exported for data quality validation using R ^2^.

Before data processing, total spectral counts for each protein and sample were normalised to the sum of spectral counts for each sample (16 samples in total, 8 biological samples and two technical replicates for each) and organism (*A. millepora, B. minutum and F. kawagutii*). Protein clusters identified in Scaffold were retained and proteins within each cluster removed from the dataset to reduce protein duplication. The mean of the two technical replicates for each sample and protein was calculated and Levene’s test used to test for equality of variances in mean spectral counts across treatments for each protein. In cases where spectral counts exhibited uneven variance across treatments (P_Leven’s_ < 0.05) the spectral counts were log10 transformed prior to test for difference between treatments using a paired t-test (n = 4, P < 0.05). The log2 fold change was calculated for each protein and proteins classified as significantly increases, decreased or unchanged based on the results of the t test and the value of the log2 fold change (negative = decrease, positive = increase, if P_t.test_ < 0.05). Finally, data quality was evaluated based on the following criteria: if a protein was indicated as decreased in abundance, it would have to be detected in at least three of the control samples, whereas if a protein was indicated as increased in abundance, it would have to be detected in at least three of the treatment samples, and finally, if no change in the relative abundance was detected, the protein would have to be present in at least three samples of both the control and treatment. Any proteins that did not comply with these requirements were excluded from further processing.

All curated proteins were annotated using the OmicsBox software (OmicsBox 1.1.78, ^12^). Each protein sequence was evaluated with the Basic Local Alignment Search Tool (BLAST) against the Swissprot database (download date, 10.05.2018; , min expectation value: 10^-5^, Number of blast hits: 5, Word size: 3, HSP Length Cutoff: 50), then processed through the successive steps for functional annotation provided by the OmicsBox (Blast2Go) software using standard settings: GO-term “Mapping”, “InterProScan” (GO-term mapping based on predicted protein structure), “Merging” of GO-terms, and finally “Annotation” where the go-terms with the highest evidence score is retained. Proteins that could not be identified using the Swissprot database were re-run against the non-redundant database (nr) using the same BLAST criteria except for a more stringent minimum expectation value (E < 10^-10^). Only unique proteins for host and symbionts based on protein names were included in the final datafiles for downstream analysis. In cases where multiple protein data rows were identified as relating to the same protein (identical name or protein id), the protein with the highest total spectral count was kept and the duplicates discarded.

Functional information for each protein was extracted from UniProt using the unique protein id obtained from the respective BLAST header. Proteins were subsequently categorised into pre-defined functional groups based on their associated GO-terms using a custom made R script, where a protein with a given GO term was assumed to be able to affect any functions linked to any *parent* of that GO term. In that way, proteins with very specific GO-terms (ex. superoxide dismutase activity: GO:0004784) were automatically grouped into higher level categories using its relationship with included parent groups (ex. antioxidant activity: GO:0016209). Finally, the functional grouping of proteins was manually curated to ensure the assumptions of the functional groups were met, and to include any proteins with no or limited GO term information that precluded automatic categorisation. For transparency, in sup. Tables 1 and 2, each functional grouping of a given protein is listed as originating from GO-term information ([GO]) or from manual categorisation ([manual]).

**Supplementary Results and discussion**

***Functional analysis of the host proteome to elevated temperature***

*Host: Energy production, respiration*

Changes to the tricarboxylic acid cycle (TCA) and electron transport system is a common response to cellular stress, whereby organisms reduce activity of pathways and reactions that produce ROS ^13^. In total, 38 proteins associated with cellular energy production in the host (glycolysis, tricarboxylic acid cycle, ATP synthesis) were detected, with the only three that increased in abundance associated with respiration; prohibitin-2 (1.52 FC) an important protein for regulation of mitochondrial degradation (as mentioned above), the electron transfer flavoprotein Alpha-ETF (1.29 FC), and NADH dehydrogenase (inf), which functions in the transfer of electrons from NADH to the respiratory chain. The relative increased abundance of these proteins could be indicative of increased cellular respiration in response to thermal stress a conserved response in many organisms ^14^. The absence of adjustment to TCA activity is counter to expectation given the other indications of cellular stress ^13^, however it is possible that TCA activity was being supported by Acetyl-CoA derived from an increase in fatty acid oxidation.

*Host: DNA maintenance, transcription and translation*

Of the 35 detected proteins involved in transcription, three increased in abundance in response to thermal stress (Fig. 4), including Prohibitin-2 (BAP-37; 1.52 FC), which regulates mitochondrial assembly and function ^15^. Prohibitin 2 has been identified as a mitophagy receptor involved in targeting mitochondria for autophagic degradation ^16^. Mitophagy (degradation of damaged mitochondria) is crucial for protecting cells against deleterious effects of damaged mitochondria and for fundamental processes in eukaryotic development. Its upregulation by the host suggests an increase in the damage rate of mitochondria, affecting the hosts ability to regenerate its energy reserves unless countered by *de-novo* mitochondria synthesis. This is supported by a previous study ^17^, in which increased degradation of host mitochondria as a result of elevated temperatures was observed in the anemone *Aiptasia pulchella*, although in another study ^18^, no significant change in the mitochondrial activity was observed in the coral *Pocillopora damicornis*. We detected 76 proteins associated with translation, of which nine were differentially expressed (Fig. 4). Of interest, we detected an increased abundance of elF3a, a protein that stimulates nearly all steps of translation initiation ^19^. The eIF-3 complex specifically targets and initiates translation of a subset of mRNAs involved in cell proliferation, including cell cycling, differentiation and apoptosis ^20^. This increased production of transcription and translation proteins is congruent with the increased need for protective chaperones such as HSPs, antioxidant enzymes and *de-novo* synthesis of damaged proteins.

***Functional analysis of the symbiont proteome to elevated temperature***

*Symbiont: DNA maintenance, transcription and translation*

We detected 71 proteins involved in the major grouping DNA maintenance, transcription and translation. There were sixteen proteins associated with transcription, of which eight increased in abundance, suggesting strong inducement of transcription processes under thermal stress. Among those proteins detected were four ATP-dependent and DEAD-box RNA helicases (1.14 - 2.8 FC), splicing factor Prp8 (0.88 FC) and U5-116kDa (1.15 FC), all essential in pre-mRNA splicing and mRNA metabolic processes ^21,22^. There was also a significant increase in the relative abundance of two ribonucleoproteins, complexes that play an integral part in several important biological functions including DNA replication, regulating gene expression and RNA metabolism. Of the 14 differentially regulated proteins associated with translation, eleven decreased in abundance (Fig. 4), the majority were parts of ribosomal subunits from 50S (chloroplastic), 60S and 40S. The three proteins that were increased in abundance were associated with the elongation and initiation steps of translation. The increased abundance of proteins involved in the regulation of gene expression and transcription processes could indicate a significant re-orientation of major cellular functions, while the more predominant decrease in translational proteins suggests a reduction in general protein production. While the former seems contradictory to the latter, a general reduction in translation could be a result of overall reduced metabolic activity concomitant with reduced photosynthetic activity and a change in the proteomic repertoire.

*Symbiont: Energy production and respiration*

Proteins associated in cellular energy production and respiration in the symbiont were mostly reduced under elevated temperature with only three proteins found to increase in abundance (Fig. 4). These included enolase (part of glycolysis), ATP synthase subunit beta (0.3 FC), and prohibitin-2 (0.4 FC), which acts as a holdase/unfoldase for the stabilisation of newly synthesised mitochondrial proteins ^23^. Key proteins of the glycolysis pathway were strongly affected, including a loss of two pyruvate kinases (PKLR, PK), and a reduction in Fructose-bisphosphate aldolase (-1.18 FC), a catalyst for gluconeogenesis. There were also reductions in the abundance of PEPCase (-0.74 FC) and PEPC 2 (-0.70 FC), a flavoprotein A3 and a Probable ATP-citrate synthase subunit 1, associated with the TCA cycle. While only two ATP synthase subunits (c and a) were affected, four proteins involved in cellular respiration decreased in abundance including cytochrome b-c1 complex Reiske-3 (-1.67 FC). These significant declines in key enzymes for glycolysis suggest reduced abundance of glucose in the system ^24^, and thus provision of sufficient substrate for maintaining high TCA activity, indicating reduced energy production and/or metabolic activity by the symbiont. The combined downregulation of TCA and respiration enzymes with photosynthetic activity reflects a strong dialling back on energy production by the cell, perhaps as a means to reduce ROS production and oxidative stress, and/or as a form of regulated reorganisation of the proteome to accommodate new environmental conditions.

*Symbiont: Cell structure and organisation, transport and signal transduction*

In stark contrast to the host proteome, there were only two proteins associated with cell structure and organisation that were differentially expressed by the symbiont, one of which was Dynein heavy chain 1 (-1.11 FC), a force-generating ATPase that moves along eukaryotic microtubules. One of the key cellular roles of the dynein family is in the movement of the axoneme – the highly conserved microtubule-based structure that provides the motility in all eukaryotic flagella and cilia ^25,26^. Reduction in this important protein may indicate poor health or considerable proteome rearrangement.

Several transporter-related proteins increased in abundance, including GTP-binding nuclear protein Ran, a GTPase involved in nucleocytoplasmic transport, participating both in the import and the export from the nucleus of proteins and RNAs ^27^. We also detected increases in the ATP-dependent RNA helicase uap56 (2.03 FC), required for the export of mRNA out of the nucleus, and in the magnesium-dependent enzyme Calcium-transporting ATPase 1 (1.76 FC), a protein that catalyses the hydrolysis of ATP coupled with the translocation of calcium from the cytosol to the endoplasmic reticulum lumen. Increased also was an ADP, ATP carrier protein (0.78). The increased abundance of this protein may serve to reduce ROS-stress by functioning as an uncoupler by dissipating the membrane potential, reducing ROS formation at the expense of ATP generation ^28^.

We detected two proteins involved in signal transduction; the downregulation (-0.68 FC) of phosphatase 2C 6, an evolutionarily conserved serine/threonine protein primarily involved in stress responses and known to reduce expression under thermal and oxidative stress, and an increase (0.45 FC) in Adenylate cyclase 1, a catalyst for the formation of signalling molecule cAMP. In *E. coli* deprived of glucose, cAMP production has been reported to increase serving as a signal to activate expression of genes for importing and metabolizing other sugars, thus the increased expression of this protein may be to induce changes in carbohydrate metabolism to support a reduction in glucose production.

1 Petrou, K., Nielsen, D. A. & Heraud, P. Single-Cell Biomolecular Analysis of Coral Algal Symbionts Reveals Opposing Metabolic Responses to Heat Stress and Expulsion. *Frontiers in Marine Science* **5**, doi:10.3389/fmars.2018.00110 (2018).

2 R Development Core Team. *R: A language and environment for statistical computing*, <<https://www.R-project.org/>> (2018).

3 Stevens, A. & Ramirez-Lopez, L. *An introduction to the prospectr package*, 2013).

4 Wagner, H., Liu, Z., Langner, U., Stehfest, K. & Wilhelm, C. The use of FTIR spectroscopy to assess quantitative changes in the biochemical composition of microalgae. *Journal of Biophotonics* **3**, 557-566, doi:10.1002/jbio.201000019 (2010).

5 Pino, L. K. *et al.* The Skyline ecosystem: Informatics for quantitative mass spectrometry proteomics. *Mass spectrometry reviews* **39**, 229-244 (2020).

6 Ying, H. *et al.* The Whole-Genome Sequence of the Coral Acropora millepora. *Genome Biology and Evolution* **11**, 1374-1379, doi:10.1093/gbe/evz077 (2019).

7 Shoguchi, E. *et al.* Draft Assembly of the Symbiodinium minutum Nuclear Genome Reveals Dinoflagellate Gene Structure. *Current Biology* **23**, 1399-1408, doi:<https://doi.org/10.1016/j.cub.2013.05.062> (2013).

8 Lin, S. *et al.* The &lt;em&gt;Symbiodinium kawagutii&lt;/em&gt; genome illuminates dinoflagellate gene expression and coral symbiosis. *Science* **350**, 691, doi:10.1126/science.aad0408 (2015).

9 LaJeunesse, T. C. *et al.* Systematic Revision of Symbiodiniaceae Highlights the Antiquity and Diversity of Coral Endosymbionts. *Current Biology* **28**, 2570-2580.e2576, doi:<https://doi.org/10.1016/j.cub.2018.07.008> (2018).

10 Keller, A., Nesvizhskii, A. I., Kolker, E. & Aebersold, R. Empirical Statistical Model To Estimate the Accuracy of Peptide Identifications Made by MS/MS and Database Search. *Analytical Chemistry* **74**, 5383-5392, doi:10.1021/ac025747h (2002).

11 Nesvizhskii, A. I., Keller, A., Kolker, E. & Aebersold, R. A Statistical Model for Identifying Proteins by Tandem Mass Spectrometry. *Analytical Chemistry* **75**, 4646-4658, doi:10.1021/ac0341261 (2003).

12 Götz, S. *et al.* High-throughput functional annotation and data mining with the Blast2GO suite. *Nucleic Acids Research* **36**, 3420-3435, doi:doi:10.1093/nar/gkn176 (2008).

13 Tomanek, L. Proteomic responses to environmentally induced oxidative stress. *The Journal of Experimental Biology* **218**, 1867, doi:10.1242/jeb.116475 (2015).

14 Brown, J. H., Gillooly, J. F., Allen, A. P., Savage, V. M. & West, G. B. Toward a metabolic theory of ecology. *Ecology* **85**, 1771-1789, doi:10.1890/03-9000 (2004).

15 Strub, G. M. *et al.* Sphingosine-1-phosphate produced by sphingosine kinase 2 in mitochondria interacts with prohibitin 2 to regulate complex IV assembly and respiration. *FASEB J* **25**, 600-612, doi:10.1096/fj.10-167502 (2011).

16 Wei, Y., Chiang, W.-C., Sumpter, R., Jr., Mishra, P. & Levine, B. Prohibitin 2 Is an Inner Mitochondrial Membrane Mitophagy Receptor. *Cell* **168**, 224-238.e210, doi:10.1016/j.cell.2016.11.042 (2017).

17 Dunn, S. R., Thomas, M. C., Nette, G. W. & Dove, S. G. A Lipidomic Approach to Understanding Free Fatty Acid Lipogenesis Derived from Dissolved Inorganic Carbon within Cnidarian-Dinoflagellate Symbiosis. *PLOS ONE* **7**, e46801, doi:10.1371/journal.pone.0046801 (2012).

18 Nielsen, D. A., Petrou, K. & Gates, R. D. Coral bleaching from a single cell perspective. *The ISME Journal*, doi:10.1038/s41396-018-0080-6 (2018).

19 Hinnebusch, A. G. eIF3: a versatile scaffold for translation initiation complexes. *Trends in Biochemical Sciences* **31**, 553-562, doi:<https://doi.org/10.1016/j.tibs.2006.08.005> (2006).

20 Lee, A. S. Y., Kranzusch, P. J. & Cate, J. H. D. eIF3 targets cell-proliferation messenger RNAs for translational activation or repression. *Nature* **522**, 111-114, doi:10.1038/nature14267 (2015).

21 Shen, J., Zhang, L. & Zhao, R. Biochemical characterization of the ATPase and helicase activity of UAP56, an essential pre-mRNA splicing and mRNA export factor. *Journal of Biological Chemistry* **282**, 22544-22550 (2007).

22 Tseng‐Rogenski, S. S. I. *et al.* Functional conservation of Dhh1p, a cytoplasmic DExD/H‐box protein present in large complexes. *Nucleic Acids Research* **31**, 4995-5002, doi:10.1093/nar/gkg712 (2003).

23 Nijtmans, L. G. *et al.* Prohibitins act as a membrane-bound chaperone for the stabilization of mitochondrial proteins. *The EMBO journal* **19**, 2444-2451, doi:10.1093/emboj/19.11.2444 (2000).

24 Burriesci, M. S., Raab, T. K. & Pringle, J. R. Evidence that glucose is the major transferred metabolite in dinoflagellate–cnidarian symbiosis. *The Journal of Experimental Biology* **215**, 3467 (2012).

25 Wickstead, B. & Gull, K. Dyneins across eukaryotes: a comparative genomic analysis. *Traffic* **8**, 1708-1721, doi:10.1111/j.1600-0854.2007.00646.x (2007).

26 Gibbons, I. R. The Role of Dynein in Microtubule-based Motility. *Cell Structure and Function* **21**, 331-342, doi:10.1247/csf.21.331 (1996).

27 Moore, M. S. & Blobel, G. The GTP-binding protein Ran/TC4 is required for protein import into the nucleus. *Nature* **365**, 661-663, doi:10.1038/365661a0 (1993).

28 Kim, E. H., Koh, E. H., Park, J.-Y. & Lee, K.-U. Adenine nucleotide translocator as a regulator of mitochondrial function: implication in the pathogenesis of metabolic syndrome. *Korean Diabetes J* **34**, 146-153, doi:10.4093/kdj.2010.34.3.146 (2010).
